# Biomolecular Condensates Can Induce Local Membrane Potentials

**DOI:** 10.1002/smll.202509591

**Published:** 2025-11-18

**Authors:** Anthony Gurunian, Keren Lasker, Ashok A. Deniz

**Affiliations:** ^1^ Department of Integrative Structural and Computational Biology The Scripps Research Institute 10500 N. Torrey Pines Rd. La Jolla CA 92037 USA

**Keywords:** biomolecular condensates, electro‐thermodynamic theory, membrane physical chemistry, membrane potential, voltage sensitive dye

## Abstract

Biomolecular condensates are a ubiquitous component of cells, known for their ability to selectively partition and compartmentalize biomolecules without the need for a lipid membrane. Nevertheless, condensates have been shown to interact with lipid membranes in diverse biological processes, such as autophagy and T‐cell activation. Since many condensates are known to have a net surface charge density and associated electric potential(s), here it is hypothesized that wetting of a membrane by a condensate can locally alter membrane potential. Using an electrochromic dye and a model system that encodes electrostatic features present in many cellular systems, it is demonstrated that poly‐lysine (polyK)/adenosine triphosphate (ATP) complex coacervates induce a localized membrane potential in Giant Unilamellar Vesicles (GUVs). This effect diminishes with increasing salt concentration and higher ATP‐to‐polyK ratios, underscoring the key role of condensate charge and corresponding Galvani potential. Numerical modeling of the condensate–membrane interface using an electro‐thermodynamic framework supports the experimental findings and highlights parameters expected to play key roles in the effect. These results have broad implications for biological processes regulated by membrane potential, particularly in contexts such as neuronal signaling, where condensate interactions with membranes may play a previously unrecognized regulatory role.

## Graphical abstract

Biomolecular Condensates are mesoscale droplets which can have a net surface charge and corresponding Donnan/Galvani Potential. Using an electrochromic dye, it is shown that, by wetting lipid membranes, condensates can alter the local membrane potential. This has significant implications for biology, where many membrane proteins are regulated by the membrane voltage.

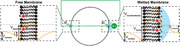

## 1 Introduction

In the last decade, biomolecular condensates have been recognized as a ubiquitous feature of cells^[^ ^1^, ^2^, ^3^ ^]^ with roles in many cellular functions ranging from nucleolar function,^[^ ^4^, ^5^, ^6^ ^]^ transcription and signaling,^[^ ^7^ ^]^ to nuclear pore permeability.^[^ ^8^ ^]^ Biological condensation has also been linked to cell misfunction and disease,^[^ ^9^ ^]^ for example through altered condensate properties,^[^ ^5^, ^10^ ^]^ protein misfolding in condensates,^[^ ^11^, ^12^, ^13^, ^14^, ^15^, ^16^, ^17^ ^]^ and viral condensates.^[^ ^18^, ^19^ ^]^ One major focus in functional studies of condensates has been their partitioning property (the ability to selectively concentrate or exclude certain molecules).^[^ ^1^, ^20^ ^]^ Partitioning of biomolecules has the effect of spatially organizing cellular processes, and has been shown to increase the rate of biomolecular reactions in some cases.^[^ ^21^ ^]^ There have also been substantial efforts in some other directions, such as condensate material, interfacial and mechanical properties, with important links to biological function and disease being uncovered.^[^ ^4^, ^5^, ^10^, ^22^, ^23^, ^24^, ^25^ ^]^ Much less is known about other properties of condensates and how they relate to biological functions.

Recently, the electrical properties of condensates have gained some attention. Electrophoretic light scattering has been used to measure condensate zeta potentials with values in the range of ‐42 mV to +42 mV.^[^ ^26^, ^27^, ^28^ ^]^ Microelectrophoresis was used to measure polylysine/polyaspartic acid condensate zeta potentials with a magnitude of 2.5 mV or 20 – 80 mV depending on which model was used to convert electrophoretic velocity to zeta potential.^[^ ^29^ ^]^ Theoretical work has shown that the interior of a condensate is charge‐neutral, while the condensate surface has a net charge resulting in electric potentials on the order of 1–10 k_B_T/e,^[^ ^30^ ^]^ which is 25–250 mV at 25 °C. Simultaneously, several authors have investigated interactions between lipid membranes and condensates including membrane wetting and endocytosis,^[^ ^26^ ^]^ membrane tubulation,^[^ ^31^ ^]^ and transmembrane coupling of condensates,^[^ ^32^ ^]^ via charge or other types of interactions. In light of these observations, we hypothesized that the wetting of membranes by condensates might alter the local membrane potential.

**Figure 1:**
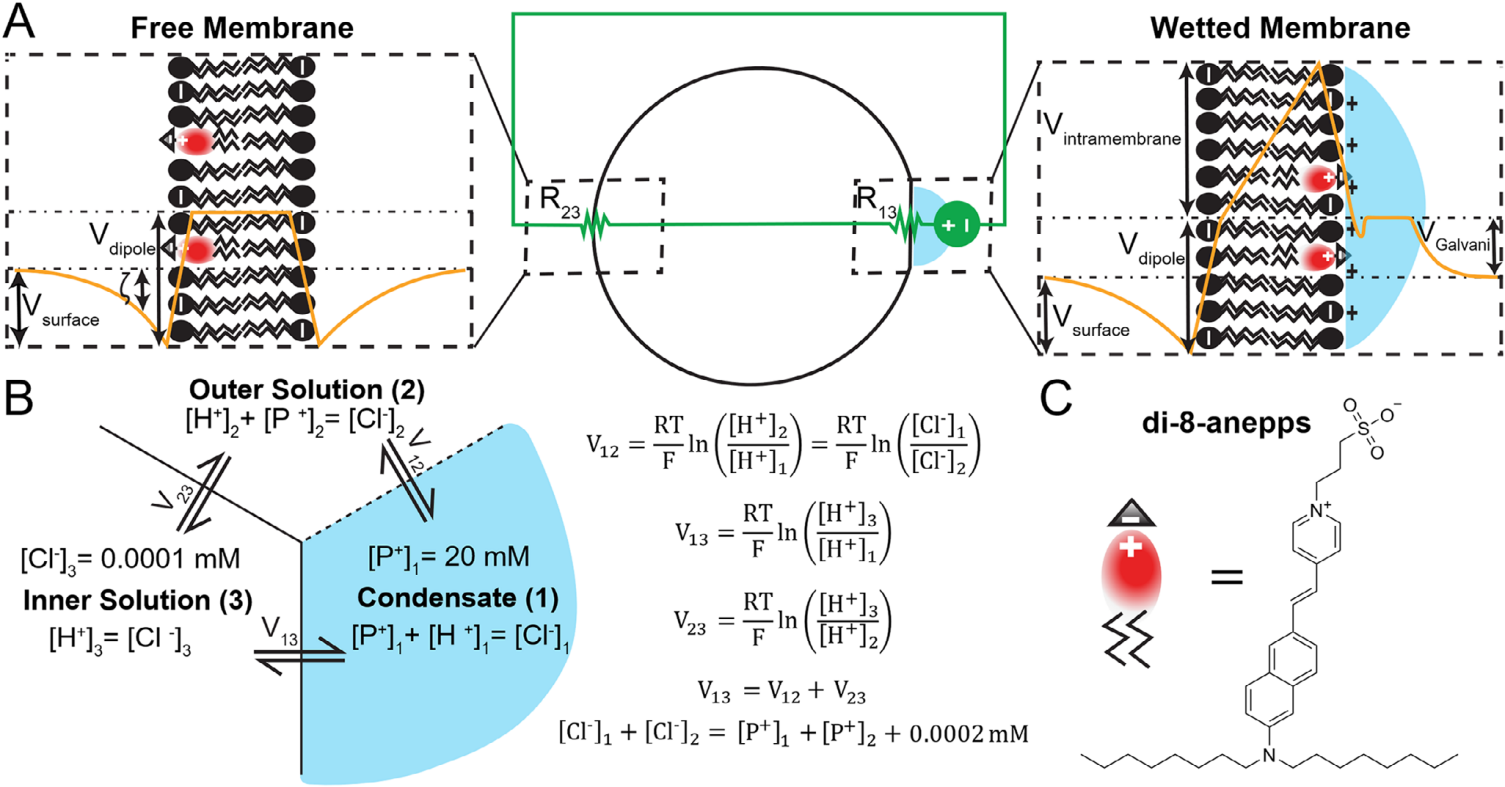
Exploratory model(s) of electric potentials associated with condensate‐membrane systems. A) Equivalent circuit representation (green) of condensate‐membrane system (middle) with resistor R_13_ corresponding to the wetted membrane region, resistor R_23_ corresponding to the free membrane region, and a voltage source representing the electric potential between the condensate and dilute phase (outer solution). Schematic illustrations (on left and right) of hypothetical electric potential profile (orange) informed by numerical simulations, across the lipid membrane (black), with associated condensate (blue), and di‐8‐anepps (red). **V_intramembrane_** – intramembrane potential, **V_surface_** – surface potential, **V_dipole_** – dipole potential, **ζ** – zeta potential, **V_Galvani_** – Galvani potential. B) Simple numerical example of 3‐compartment Donnan‐type equilibrium with P^+^ as an impermeable cation, Cl^−^ as the counterion. Dashed line is permeable to H^+^ and Cl^−^, and solid lines are permeable to H^+^ but not Cl^−^. Right: Equations for Nernst equilibrium of permeable ions and Kirchoff's Voltage Law. C) Chemical structure of 4‐[2‐[6‐(dioctylamino)‐2‐naphthalenyl]ethenyl]‐1‐(3‐sulfopropyl)‐pyridinium (di‐8‐anepps).

Consider the system consisting of a Giant Unilamellar Vesicle (GUV) wetted by a condensate (**Figure** **1**). To get a quick intuition about the system, we constructed an equivalent circuit model. We modeled the condensate dense/dilute phase interface as a voltage source, whose magnitude corresponds to the condensate Galvani potential. The wetted membrane region and free membrane region are modeled as resistors with resistance R_13_ and R_23_, respectively. Immediately, we see that, due to Kirchoff's Voltage Law, the electric potential which develops across the condensate dense/dilute phase interface gets transferred across the membrane (R_13_ and R_23_). Next, to get a quantitative and more thermodynamically sound picture of the system, we considered the system as a simplified 3‐compartment Donnan‐type equilibrium (Figure 1B). The condensate consists of an impermeable cation P^+^ (which could represent a condensing protein) at a fixed concentration, chloride (Cl^−^) as the counterion, and is in equilibrium with the dilute phase (outer solution) and the GUV lumen (inner solution). The system obeys the usual Nernst equilibrium equations, Kirchoff's Voltage Law, and electroneutrality equations. Solving the system using realistic numerical values (Figure 1B), we find that, in the limit of high partition coefficient (for [P^+^]_2_ ≈ 0), V_12_ = 295.8 mV, V_13_ = 278.0 mV, and V_23_ = ‐17.8 mV. This means that most of the voltage (94% in this example) drops across the wetted‐membrane region (R_13_), resulting in a local difference in membrane potential. We also determined the general solution as a function of the effective dense‐dilute phase partition coefficient by varying [P^+^]_2_ while holding [P^+^]_1_ constant, showing that the previously noted intuition holds for a range of reasonable effective partition coefficient (see Note S1 (Supporting Information) for more detailed description; Table S1 and Figure S1 (Supporting Information) for results).

Succinctly, our hypothesis is that biomolecular condensates can induce local membrane potentials. This could be primarily as a result of the condensate Galvani potential (as described above), which results in a local transmembrane potential (V_TM_) and consequently an (intra)membrane potential (V_intramembrane_). In addition, intramembrane potentials are also thought to arise as a result of *asymmetric* surface potentials (V_surface_) or *asymmetric* dipole potentials (V_dipole_),^[^ ^33^, ^34^, ^35^ ^]^ which could also contribute to the total intramembrane potential in our system (Figure 1A). In classical electrophysiology, the value of the intramembrane potential is usually assumed to be equivalent to that of the transmembrane potential which arises due to asymmetric ion concentrations following the Goldman‐Hodgkin‐Katz (GHK) equation.^[^ ^36^ ^]^ Recently, the electrochromic dye di‐4‐anepps was used to show *global* changes in membrane potential in E. coli due to global changes in intracellular ion concentration resulting from condensate formation.^[^ ^37^ ^]^ However, since ions are free to diffuse in all directions, it should be highly disfavored to have any kind of *local* difference in membrane potential at equilibrium within the framework of the GHK equation. Here, we use di‐8‐anepps, an electrochromic dye, to show that condensates can alter the *local* membrane potential even in the absence of ion channels or pore forming peptides. This could have large implications for the regulation of voltage gated ion channels and other electric field sensitive membrane proteins, which we elaborate on in the discussion.

## 2 Results and Discussion

### 2.1 Condensates can Induce Local Membrane Potentials

To evaluate whether condensates can induce membrane potentials, we used a model system which encodes key physicochemical properties present in many cellular systems. Our system consisted of GUVs (Giant Unilamellar Vesicles) composed of 1‐palmitoyl‐2‐oleoyl‐sn‐*glycero*‐3‐phosphocholine (POPC) and 1,2‐dioleoyl‐sn‐glycero‐3‐phosphatidic acid (PA), mixed with poly‐lysine (polyK)/adenosine triphosphate (ATP) complex coacervates. The GUVs provide a roughly cell‐sized system that incorporates the generally negatively charged character of cell membranes, with PA also being involved in several biological activities.^[^ ^38^ ^]^ Additionally, positively charged lysine‐rich protein sequences are often important in condensates,^[^ ^39^, ^40^, ^41^ ^]^ and ATP is ubiquitous biomolecule in cells which has been shown to modulate condensate properties.^[^ ^42^, ^43^ ^]^ We stained the GUV membranes with an electrochromic dye, di‐8‐anepps,^[^ ^44^ ^]^ which shifts its excitation and emission spectra in the presence of a local electric field. The shift in absorption frequency can be described by the equation:

(1) $$h\Delta v=-\left(\Delta \vec{\mu }\right)\cdot \vec{E}=-\left|\Delta \mu \right|\left|E\right|\cos\theta$$

where *h* is Planck's constant, *E* is the local electric field strength, and $\Delta \vec{\mu }$ is the change in dipole moment between the ground state and the excited state, and θ is the angle between $\Delta \vec{\mu }$ and electric field vector $\vec{E}$.^[^ ^45^, ^46^ ^]^ Following previous work,^[^ ^44^ ^]^ we use the excitation ratio R_440/514_ to quantify the shift in the excitation spectrum. Compared to absolute intensity measurements, the ratiometric approach has the advantage of being independent of local variations in dye concentration. Also, we chose di‐8‐anepps because of its very low rate of flipping between leaflets of the lipid bilayer.^[^ ^47^ ^]^ In the following experiments, we assume that most of the dye is retained in the outer leaflet during the experiment.

**Figure 2:**
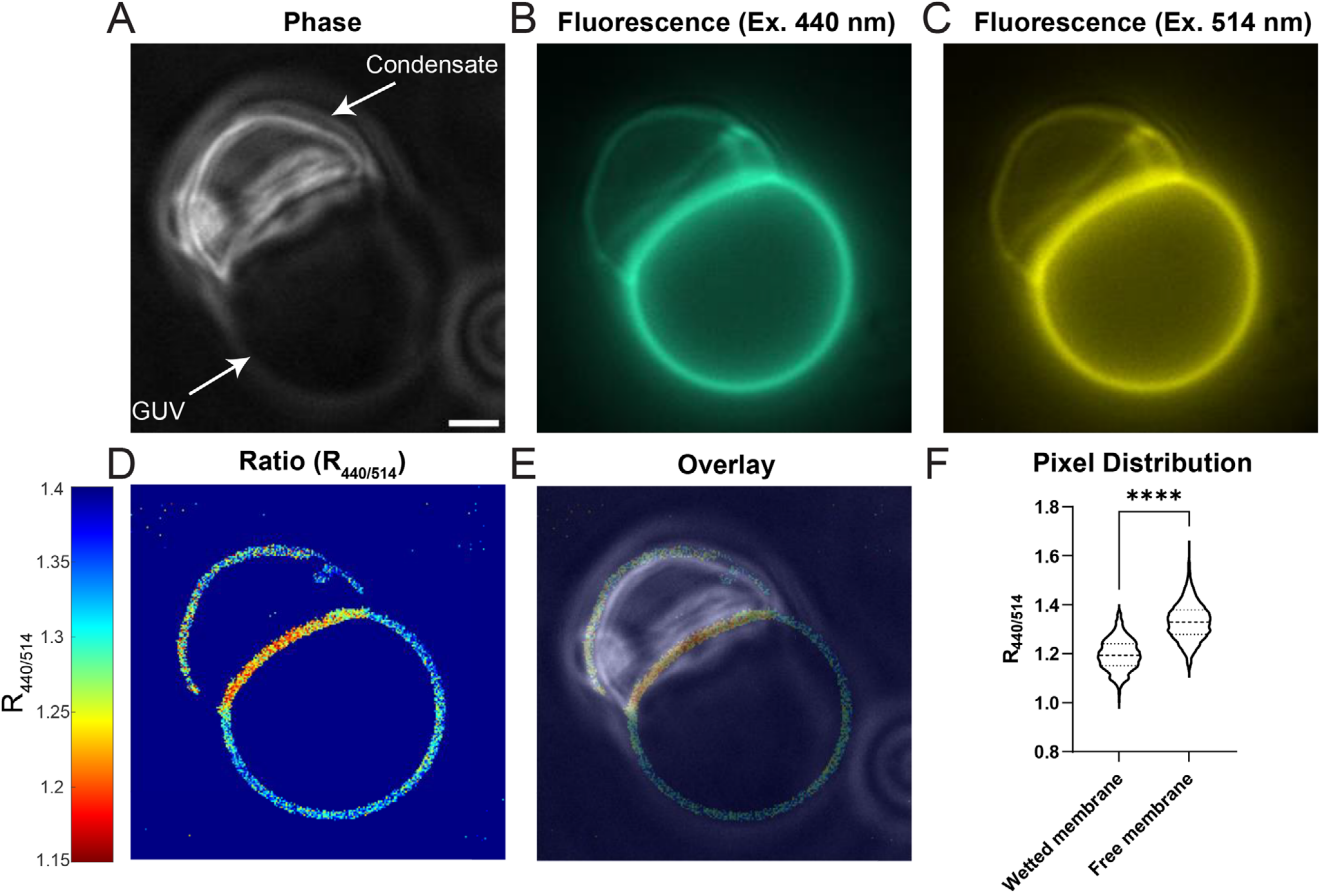
Fluorescence imaging demonstrates condensate induced local membrane potential. GUVs composed of 80% POPC, 20% PA are wetted by polyK/ATP condensates (20 µM polyK‐50, 20 µM polyK‐100, 1 mM ATP, 2.5 mM MgCl_2_, 500 mM glucose, 50 mM Tris‐HCl pH 7.5). A) Phase contrast image of condensate wetting GUV. B,C) Fluorescence images of GUV membrane labeled by di‐8‐anepps, with 440 nm and 514 nm excitation respectively. D) Thresholded raw ratiometric image. E) Overlay of phase contrast image and ratiometric image. F) Distribution of background subtracted pixel intensity ratios in wetted membrane segment and free membrane segment (n = 651, 1778). Statistical analysis is unpaired t‐test with Welch's correction, p < 0.0001,^****^, Scale Bar 2 µm.

By mixing GUVs composed of zwitterionic POPC and anionic PA, with positively charged polyK /ATP complex coacervates, we observed membrane wetting and changes in curvature (**Figure** **2**A–C). We also observed a striking local red shift of the dye excitation ratio R_440/514_, indicating a local change in membrane potential (Figure 2D–F; Figures S2 and S12, Supporting Information). According to our model (Figure 1A), the adsorption of the positively charged condensate outside the GUV creates an electric field directed toward the inside of the GUV. Since the dye is oriented with the sulfonate group pointing outward (Figure 1), the electric field vector is parallel to the vector $\Delta \vec{\mu }$,^[^ ^46^ ^]^ resulting in a negative change in frequency, or red shift, according to Equation (1), in agreement with our result. Note that alternatively, if the condensate has the effect of lowering the membrane dipole potential (V_dipole_), this would also result in a red shift since the electric field due to the dipole potential is directed antiparallel to the vector $\Delta \vec{\mu }$.

### 2.2 The Induced Effects are Directly Related to Condensate Charge, Along with Other Contributions

**Figure 3:**
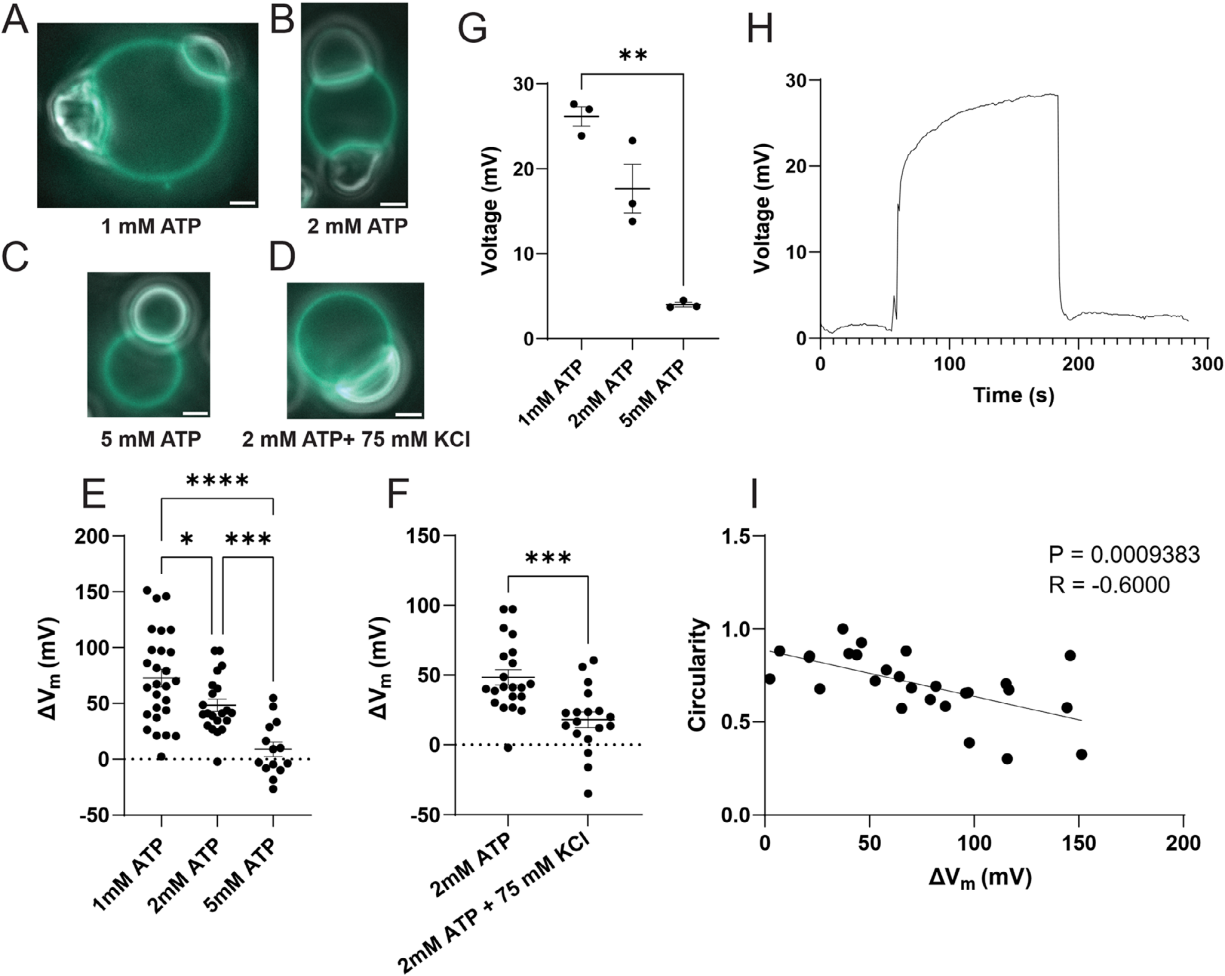
Experimental parameters which influence condensate‐induced membrane potential A–D) Representative images (Overlay of Fluorescence (440 nm) and Phase Contrast) (A) 1 mM ATP (B) 2 mM ATP (C) 5 mM ATP (D) 2 mM ATP + 75 mM KCl. GUVs are composed of 80% POPC, 20% PA, with polyK/ATP condensates (20 µM polyK‐50, 20 µM polyK‐100, x mM ATP, 2.5 mM MgCl_2_, 500 mM glucose, 50 mM Tris‐HCl pH 7.5). E) Summary figure of ΔV_m_ as a function of ATP concentration (n = 27, 21, 14). Statistical analysis is One‐way ANOVA and Dunnett's T3 multiple comparisons test (p<0.0001,^****^ | p<0.001,^***^ | p<0.05,*). F) Effect of Salt on ΔV_m_ (n = 21, 18). Statistical analysis is unpaired t‐test with Welch's correction (p < 0.001,^***^) G) Summary figure of condensate Galvani Potential measured using glass capillary electrode with polyK/ATP condensates (20 µM polyK‐50, 20 µM polyK‐100, x mM ATP, 2.5 mM MgCl_2_, 500 mM glucose, 50 mM Tris‐HCl pH 7.5). Statistical analysis is One‐way ANOVA and Dunnett's T3 multiple comparisons test (p<0.01,^**^). H) Representative voltage recording of condensate Galvani Potential corresponding to (20 µM polyK‐50, 20 µM polyK‐100, 1 mM ATP, 2.5 mM MgCl_2_, 500 mM glucose, 50 mM Tris‐HCl pH 7.5). I) Correlation of condensate circularity with ΔV_m_ (n = 27). Condensates (20 µM polyK‐50, 20 µM polyK‐100, 1 mM ATP, 2.5 mM MgCl_2_, 500 mM glucose, 50 mM Tris‐HCl pH 7.5). R value is Pearson's correlation coefficient. Scale Bar 2 µm. Error bars represent SEM.

To demonstrate that our observed effect is directly related to the charge of the condensate, we first titrated the concentration of ATP. Increasing the concentration of ATP should decrease the magnitude of the condensate surface charge density, by reducing the polyK/ATP ratio.^[^ ^29^ ^]^ To confirm this assumption, we spun down 10 mL solutions of polyK/ATP condensates and directly measured the Galvani potential using glass capillary electrodes (**Figure** **3**H). Figure 3G shows that decreasing the polyK/ATP ratio decreased the magnitude of the Galvani potential. Figure 3A,B,C,E shows that the less positively charged coacervates wet the membrane to a lesser extent, and as expected based on the model, induce a lower membrane potential. Next, we performed another control, where we increased salt concentration, which should screen the condensate interactions with the membrane, and lower the condensate Galvani potential.^[^ ^30^ ^]^ Figure 3D,F shows exactly this trend. These control experiments strongly support the (expected) major role of condensate charge on the induced local membrane potentials.

We wondered what other characteristics of condensates might affect the magnitude of the change in membrane potential (Δ*V_m_*), and whether they may be responsible for the large standard deviations in our measurements of membrane potential (Figure 3E, F). Throughout the course of our experiments, we noticed that sometimes within the same condition (e.g., Figure 3A,B; Figure S14, Supporting Information) there were coexisting populations of spherical, liquid‐like condensates, and irregularly shaped, gel‐like condensates. Using FRAP (fluorescence recovery after photobleaching), we observed slower recovery, which should correlate with higher viscosity, for irregularly shaped (versus circular) condensates (Figure S3, Supporting Information). We then measured the condensate geometric parameters (circularity, solidity, roundness, aspect ratio; see Figure S4, Supporting Information for definitions) and found moderate correlations with Δ*V_m_* (Figure 3H; Figures S4 and S5, Supporting Information); the correlation with circularity (4π×Area/perimeter^2^; Figure 3H) was particularly good (r = ‐0.6, p < 0.001), suggesting that both shape elongation and irregularities of the shape boundary are important. Since the shape of a condensate changes when it wets a lipid membrane, with less round shapes and higher aspect ratios typically reflecting greater wetting and stronger interactions,^[^ ^26^ ^]^ some of our observed correlations may be simply because condensates with a greater charge have a stronger interaction with the membrane.

To further generalize our findings, we used an additional system consisting of poly(RG)/ATP condensates and were able to recapitulate our results (Figure S6, Supporting Information). Of note, RG or RGG repeats are a common motif in biologically relevant phase separating proteins, including FUS, hnRNPA1, TDP‐43, and G3BP1.^[^ ^48^, ^49^, ^50^, ^51^ ^]^

Together, our experimental results demonstrate the induction of local membrane potentials by interacting condensates. Our results also directly show the key role of condensate charge in this effect, with other condensate parameters having an influence.

### 2.3 Analytical Framework and Simulations to Explore Qualitative Features of the Induced Potentials

**Figure 4:**
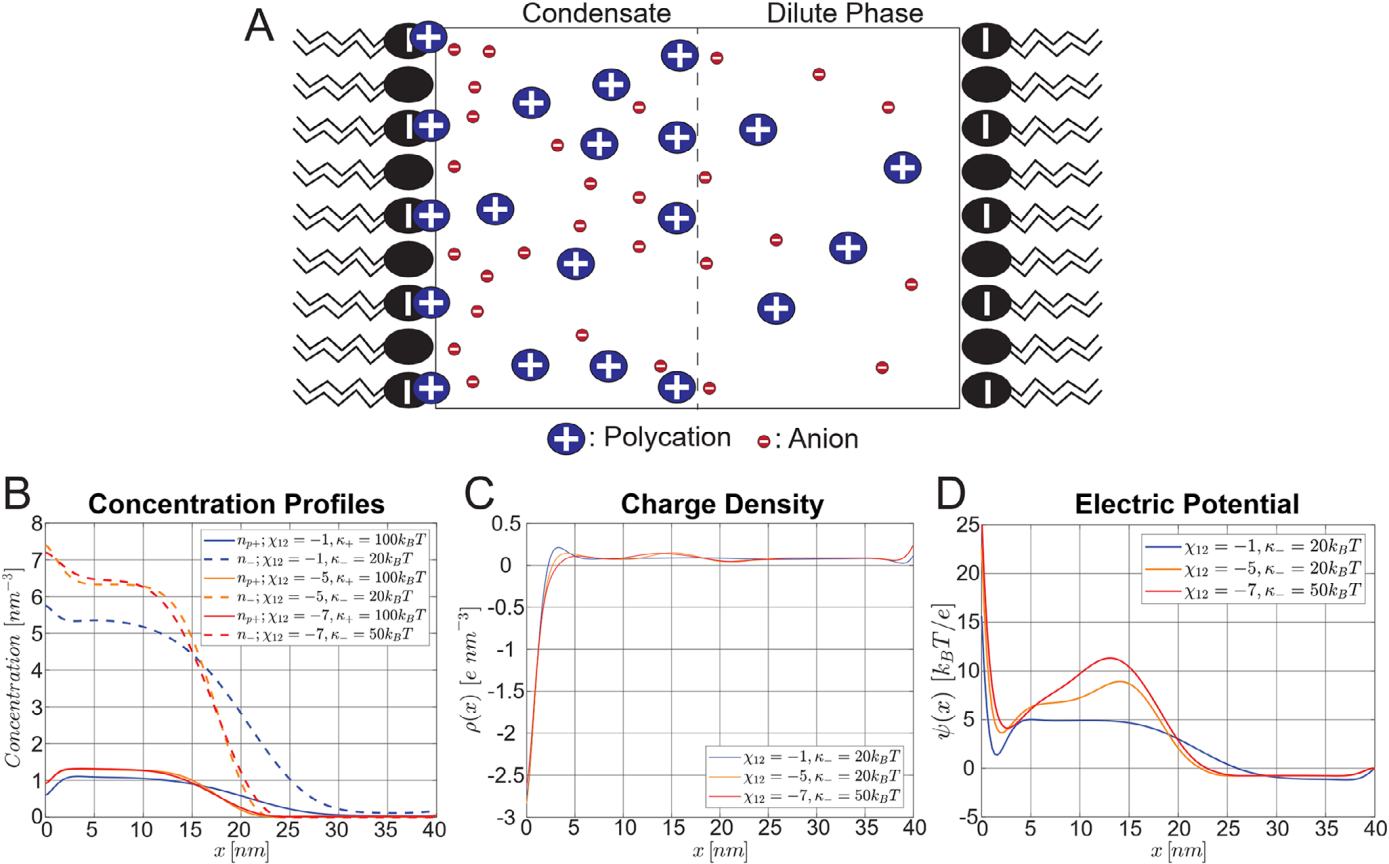
Numerical model of a condensate in the presence of charged membranes. (p+) indicates the polycation while (‐) indicates the anion. Parameters used for calculation: κ_*p* +_ = 100*k_B_T*;  κ_−_ = 20*k_B_T*;  χ_13_ = 1.5;  χ_23_ = −0.09; *N*_1_ = 20; *N*_2_ = 1; *N*_3_ = 1;  *v* = 0.03 *nm* ^3^; z_1_ =   5; z_2_ =   − 1; z_3_ = 0;  *A_L_* = 0.5 *nm* ^2^; *f* = 0.5;  *K*_*p* +_ = 5. Definitions: κ_*i*_ – gradient cost for component *i*, χ_*ij*_ – Flory‐Huggins interaction parameter between components *i* and *j*, *N_i_* – degree of polymerization for component *i*, *v* – molecular volume of monomer, z_*i*_ – valence of component *i*, *A_L_* – area per lipid molecule, *f*– fraction of charged lipids, *K*_*p* +_ – p^+^‐membrane binding constant. A) Schematic illustration of model geometry B) Equilibrium concentration profiles. Region with high concentrations corresponds to the dense phase (condensate), region with low concentrations corresponds to the dilute phase. C) Equilibrium charge density. Separation of charges leads to electric potential. D) Equilibrium Electric Potential.

Finally, to complement our experimental work and provide additional qualitative mechanistic insights into our observed effects, we developed an analytical theory framework and used it to carry out numerical calculations to determine the theoretical electrical potential profile at the membrane‐condensate interface. We build off an existing model which couples the Poisson equation with a free energy functional combining the Flory‐Huggins free energy with electrostatic free energy and concentration gradient terms,^[^ ^30^, ^52^ ^]^ and ask what happens in the presence of a charged membrane. **Figure** **4**A shows a schematic illustration of our model geometry and results. The GUV membrane is modeled as an idealized charged surface, and is implemented in the model using the boundary conditions $\frac{d\psi }{dx}{|}_{x=0}=-\frac{\sigma }{\varepsilon };\;\frac{d\psi }{dx}{|}_{x=N}=\frac{\sigma }{\varepsilon }$, where ψ is the electric potential, σ is the membrane surface charge density, and ε is the permittivity, with the wetted membrane corresponding to x = 0, and the free membrane corresponding to x = N (see Note S2, Supporting Information for details). The electric potential at the wetted membrane (Figure 4D, x = 0 nm) is approximately the sum of the condensate Galvani potential which develops between the dense and dilute phase, and the surface potential resulting from the adsorption of polycations (p^+^) to the negatively charged membrane. Note that in these simulations, the potential at the wetted membrane (Figure 4D, x = 0 nm) is relative to the potential at the free membrane (Figure 4D, x = 40 nm). Since we used the Langmuir Isotherm to model polycation binding, in which the fraction of bound sites is a hyperbolic function of the local polycation concentration, more binding occurs at the wetted membrane due to the higher polycation concentration in the condensate. Anions accumulate immediately adjacent to the membrane (Figure 4B), resulting in a net negative charge density (Figure 4C), and a potential well (Figure 4D, x = 2 nm). For the case where the Flory‐Huggins interaction parameter is χ_12_ = −5 or − 7, with a more negative value of χ_12_ indicating a stronger interaction between the coacervating components, there is also an interfacial component to the electric potential between the dense and dilute phases (Figure 4D) which has been observed previously.^[^ ^30^ ^]^ Together, this results in a polarization of the condensate, and a non‐negligible electric field $\left(\vec{E}=\frac{d\psi }{dx}\approx 10^{7}Vm^{-1}\right)$ within the bulk of the condensate. This could have implications for the conformation of client proteins within the condensate, which would tend to align their net dipole moments with the electric field. However, we emphasize that our simulation corresponds to a condensate on the length scale of tens of nanometers, and the effect may be different for larger condensates. We also analyzed the effect of lowering the p^+^‐membrane binding constant (K_p+_) and found that for weak binding (K_p+_ = 0.01), the surface potential is negative and thus lowers the total potential at the wetted membrane (Figure S7, Supporting Information, panel D). Overall, our model and simulations provide additional insight into the nanoscale profile of the electric field at the condensate‐membrane interface and makes the prediction that condensates with a stronger interaction parameter χ_12_ and p^+^‐membrane binding constant (K_p+_) will result in a larger difference in membrane potential (Figure 4D; Figure S7, Supporting Information).

### 2.4 Overall Considerations and Biological/Chemical Implications

We have shown for the first time that it is possible to generate local differences in membrane potentials at equilibrium. Traditionally, the cell has often been assumed to be analogous to a membrane bound vesicle containing a dilute solution of ions, proteins, and nucleic acids. Since accumulating ions in localized regions is highly disfavored due to the large entropic cost, the concept of local membrane potential has been largely neglected, except for the well‐known case of the action potential, which is transient. Condensates, due to their electrical properties (Galvani potential), and the property of being spatially segregated units, are able to induce local membrane potentials where they come into contact with, and wet, lipid membranes.

We mentioned that changes in dipole potential or surface potential may also contribute to our observed results (Figure 1A). Since these changes are presumably asymmetric (only on one side of the membrane), they will also result in an intramembrane potential.^[^ ^33^, ^34^, ^35^ ^]^ A recent paper used LAURDAN, a solvatochromic dye, to show condensate induced local changes in membrane *hydration*.^[^ ^53^ ^]^ Changes in the average orientation, concentration, or rotational dynamics of water molecules at the membrane‐condensate interface could result in an altered membrane dipole potential. Note that since this dipole potential occurs over such a short distance, it has a larger electric field associated with it (E = V/d), so di‐8‐anepps is particularly sensitive to the dipole potential. Previous observations of local variations of membrane potential were all in cells and have been typically attributed to local variations in dipole potential due to variations in lipid composition.^[^ ^54^, ^55^ ^]^ We should also note that others have recently hinted at the converse of our observation, that variations in the dipole potential may drive condensate association with certain membrane regions.^[^ ^56^ ^]^

We considered the possibility of lipid demixing and accumulation of phosphatidic acid (PA) near the condensate as an alternative explanation for our results. However, the PA headgroup was previously shown to increase the dipole potential and R_420/520_,^[^ ^57^, ^58^ ^]^ whereas we see a decrease at the wetted membrane region. We also considered whether the different lipid tail structure (18:1 PA versus 16:0–18:1 PC), which is related to the dipole potential via lipid packing,^[^ ^59^ ^]^ could be contributing to our results. We did control experiments with DOPC/DOPA GUVs (18:1 PC, 18:1 PA) and POPC/POPS (1‐palmitoyl‐2‐oleoyl‐sn‐glycero‐3‐phospho‐L‐serine^[^ ^60^ ^]^) GUVs (16:0–18:1 PC, 16:0–18:1 PS) and were still able to recapitulate our results, indicating our results are reproducible across a range of lipid tail and headgroup structures (Figures S8 and S9, Supporting Information). Our observations are also not due to a condensate induced increase in lipid packing,^[^ ^53^ ^]^ since this would also cause an increase in R_420/520_.^[^ ^59^ ^]^

Ermakov and coworkers have extensively studied the interaction of polyK with lipid membranes.^[^ ^61^, ^62^ ^]^ Using zeta potential measurements of liposomes, they observed a sudden, sharp increase in zeta potential at a lysine (monomer) concentration of approximately 200 µM, which corresponds to 2 µM polyK‐100. In terms of our results, if the condensate (dense phase) concentration is higher than this cutoff, and the dilute phase concentration is less than the cutoff, then our observed differences in membrane potential could also be explained by differences in surface potential. While our model suggests polyK also adsorbs to the membrane adjacent to the dilute phase (Figure 4), we were unable to see evidence (within our experimental sensitivity) of polyK adsorption to free membrane regions using condensates doped with labeled polyK‐Atto565 (Figure S10, Supporting Information), and in similar systems used by others.^[^ ^26^ ^]^ Importantly, polyK‐Atto565 by itself does adsorb to GUVs in the absence of ATP (Figure S10, Supporting Information). Interestingly, like the wetted membrane, the free membrane also shows shifts in R_440/514_ as a function of ATP concentration (Figure S11, Supporting Information), suggesting that dilute phase‐membrane interactions might also produce some voltage change, consistent with the abovementioned model‐predicted polyK binding to the membrane. We added polyK alone to POPC/PA vesicles stained with di‐8‐anepps, and found that the value of R_440/514_ decreases with increasing polyK concentration (Figure S13, Supporting Information), suggesting that our observed effect is at least partly related to changes in surface and/or dipole potential. A similar effect was seen previously with POPC/POPG (phosphatidylglycerol) SUVs (small unilamellar vesicles), but only with high molecular weight (>70 kDa) poly‐lysine.^[^ ^63^ ^]^

In addition, using the intramembranous field compensation (IFC) method with planar lipid bilayers, Ermakov and coworkers observed that changes in boundary potential upon titration with lysine were right shifted compared to changes in zeta potential, and attributed this to opposite changes in the dipole potential.^[^ ^61^, ^62^ ^]^ Using molecular dynamics simulations, they showed that lysine molecules displace water molecules which previously made hydrogen bonds with lipid phosphate groups, and since water molecules are thought to be responsible for the dipole potential,^[^ ^64^ ^]^ it was concluded that changes in the number and/or orientation of water molecules is responsible for the decrease in dipole potential.^[^ ^61^, ^62^ ^]^

**Figure 5:**
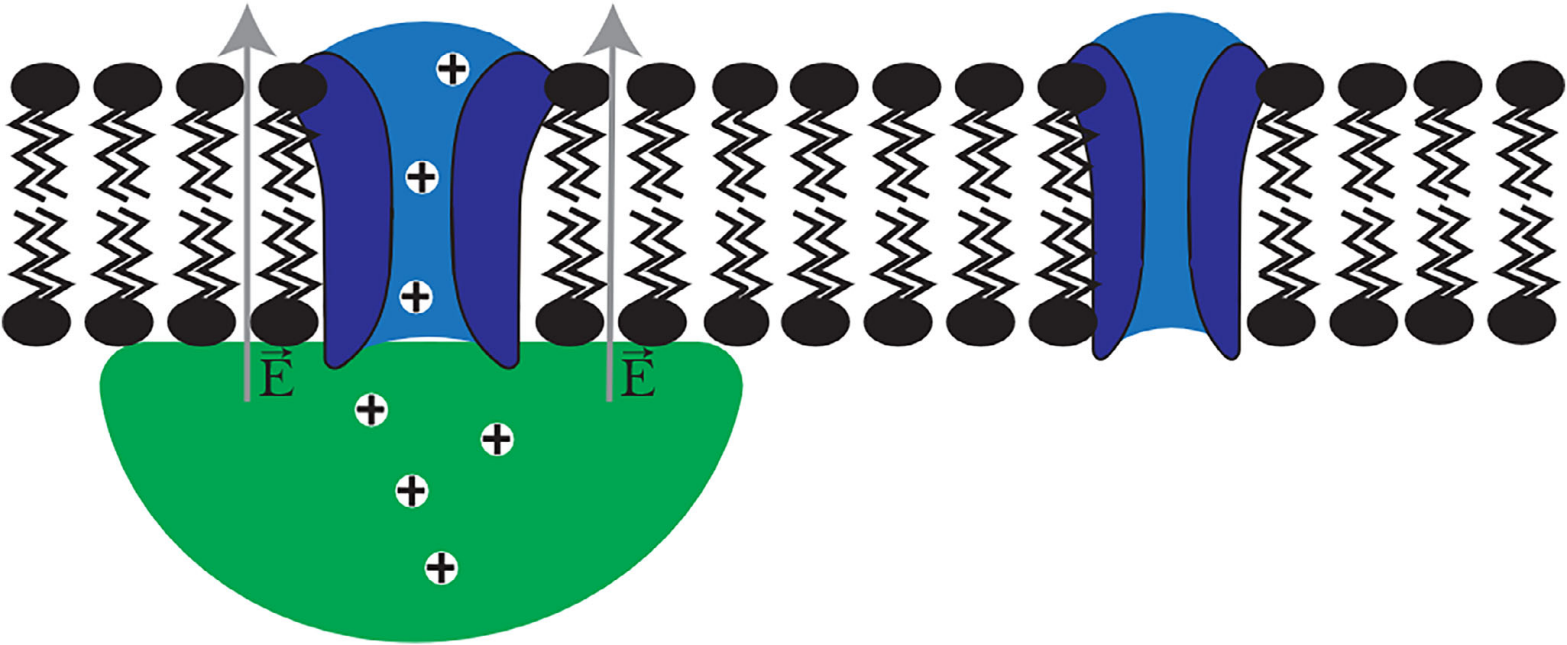
Biological implications and Future Directions. Illustration of hypothetical activation of voltage gated ion channels (blue) by condensates (green) due to local induced membrane potential.

Local changes in membrane potential (intramembrane or dipole potentials^[^ ^65^, ^66^, ^67^, ^68^ ^]^) could have large implications for the function of ion channels and other transmembrane proteins which are regulated by voltage. For example, voltage‐gated ion channels change their conformation depending on the membrane potential and consequently control the flux of ions in and out of the cell.^[^ ^68^ ^]^ Therefore, local differences in membrane potential could facilitate localized flux of ions, permitting precise spatiotemporal control over cell signaling (**Figure** **5**). On a different level, the basic equations for the flux of ions should be modified to account for the presence of biomolecular condensates. The flux of ions has traditionally been given as the sum of the diffusive flux determined by the concentration gradient, and the electrophoretic flux determined by the voltage gradient.^[^ ^36^ ^]^ In the presence of condensates, this should be modified to include a chemical potential gradient $\frac{\partial \mu }{\partial x}$ due to the differential partitioning of specific ions in condensates,^[^ ^69^, ^70^ ^]^ and a modified voltage gradient $\frac{\partial V_{m,c}}{\partial x}$ taking into account the condensate induced membrane potential. We can express the modified flux as,

(2) $$J=-D\frac{\left[C\right]}{RT}\frac{\partial \mu }{\partial x}-mz\left[C\right]\frac{\partial V_{m,c}}{\partial x}\;$$

where D is the diffusion coefficient, [*C*] is the ion concentration, R is the gas constant, T is the temperature, m is the ion mobility, and z is the ion valence. One interesting example of a condensate associated with ion channels is the RIM/RIM‐BP condensate which clusters voltage‐gated calcium channels (VGCCs) at the neuronal pre‐synapse.^[^ ^71^ ^]^ In future work, we anticipate examining whether this condensate can regulate VGCCs by inducing a membrane potential.

Finally, we can also connect our results to two other conceptual directions in the condensate field. In one, recent work has demonstrated the ability of condensate surfaces (and associated potentials) to alter the kinetics of covalent chemical reactions such as the hydrolysis of ATP.^[^ ^72^, ^73^ ^]^ We speculate that similar effects might occur within or adjacent to the membrane regions perturbed by interactions with charged condensates, an exciting direction we aim to pursue in future work. In another, we note that while we only explored the role of relatively large micron‐scale condensates in this work, our findings should also apply to interactions of nanoscale charged condensates (e.g., percolation clusters^[^ ^74^, ^75^, ^76^ ^]^) with membranes, as also suggested by our simulations. Thus, exploring for induced local membrane potentials and their biological consequences in such a context (for example, in the synaptic region) is another interesting future direction.

## 3 Conclusion

In conclusion, we have shown that biomolecular condensates can induce local membrane potentials when they wet lipid membranes. We showed that the effect is directly related to the condensate charge, but more work is needed to understand the precise mechanism. Future work will also explore the possible role of condensates in regulating membrane proteins sensitive to electric potential and a possible role of the discovered effect on local modulation of chemical reactions.

## 4 Experimental Methods

Detailed experimental methods for Sample Preparation, Imaging and Analysis, FRAP, polyK labeling and materials were provided in the Supporting Information.

The authors have cited additional references within the Supporting Information.^[^ ^77^, ^78^, ^79^ ^]^

## Conflict of Interest

The authors declare no conflict of interest.

## Supporting information



Supporting Information

## Data Availability

The data that support the findings of this study are openly available in Zenodo at https://doi.org/10.5281/zenodo.14968978, reference number 14968978.
