# Supplementary material for: Biomolecular Condensates Can Induce Local Membrane Potentials
Source: Small. 2025 Nov 18;22(1):e09591. doi: 10.1002/smll.202509591 (PMC12757988; doi:10.1002/smll.202509591)
Supplement: Supplementary file 1 — Supporting Information [file SMLL-22-e09591-s001.pdf]

NANO · MICRO
small

Supporting Information

for *Small*, DOI 10.1002/smll.202509591

Biomolecular Condensates Can Induce Local Membrane Potentials

*Anthony Gurunian, Keren Lasker and Ashok A. Deniz**

Biomolecular Condensates can Induce Local Membrane Potentials

SUPPORTING INFORMATION

(Supplementary Figures, Notes, Table & Materials and Methods)

Anthony Gurunian, Keren Lasker & Ashok A. Deniz

Department of Integrative Structural and Computational Biology, The Scripps Research Institute, 10550 N. Torrey Pines Rd., La Jolla, CA 92037

Contents

Supplementary Note S1: Simple Donnan Equilibrium Model 3

Supplementary Table S1: Solution to Donnan Equilibrium model in the limit of high partition coefficient 4

Figure S1: General solution to Donnan Equilibrium model. 5

Supplementary Note S2: Analytical Framework 6

Figure S2: Main-text Figure 2 without thresholding. 8

Figure S3: FRAP (Fluorescence Recovery After Photobleaching) measurements. 9

Figure S4: Correlation of condensate shape parameters with ΔV_m 10

Figure S5: Condensate shape parameters across different ATP concentrations. 11

Figure S6: poly(RG)/ATP condensate – GUV measurements. 12

Figure S7: Effect of varying binding constant K_{p+} in numerical simulation of a condensate in the presence of a charged membrane. 13

Figure S8: DOPC/PA GUV control. 14

Figure S9: POPC/POPS GUV control. 15

Figure S10: Localization of labeled polyK-Atto565. 16

Figure S11: $R_{440/514}$ values for membrane regions used to calculate ΔV_m in Figure 3. 17

Figure S12: Electroformation control. 18

Figure S13: polyK titration. 19

Figure S14: Full fields of view. 20

Supplementary Materials and Methods 21

SI References 24

Supplementary Note S1: Simple Donnan Equilibrium Model

Consider a 3-compartment Donnan equilibrium (Figure 1B). The condensate consists of an impermeable cation P^+ (which could represent a condensing protein) at a fixed concentration, chloride (Cl^-) as the counterion, and is in equilibrium with the dilute phase (outer solution) and the GUV lumen (inner solution). Hydrogen ions (H^+) are also present in the system. The condensate dense/dilute phase interface is permeable to both H^+ and Cl^- while the GUV membrane is only permeable to H^+. The governing equations of the system are the Nernst equilibrium equations,

$$V_{12} = \frac{RT}{F} \ln\left(\frac{[H^+]_2}{[H^+]_1}\right) = \frac{RT}{F} \ln\left(\frac{[Cl^-]_1}{[Cl^-]_2}\right) \tag{1}$$

$$V_{13} = \frac{RT}{F} \ln\left(\frac{[H^+]_3}{[H^+]_1}\right) \tag{2}$$

$$V_{23} = \frac{RT}{F} \ln\left(\frac{[H^+]_3}{[H^+]_2}\right) \tag{3}$$

Kirchoff's Voltage Law,

$$V_{13} = V_{12} + V_{23} \tag{4}$$

and the electroneutrality equations,

$$[P^+]_1 + [H^+]_1 = [Cl^-]_1 \tag{5}$$

$$[P^+]_2 + [H^+]_2 = [Cl^-]_2 \tag{6}$$

$$[H^+]_3 = [Cl^-]_3 \tag{7}$$

We fix the impermeable cation concentrations in the dense phase,

$$[P^+]_1 = 20\, mM \tag{8}$$

and provide sufficient Cl^- counterions,

$$[Cl^-]_1 + [Cl^-]_2 = 20.0002\, mM + [P^+]_2 \tag{9}$$

$$[Cl^-]_3 = 0.0001\, mM \tag{10}$$

which accounts for the presence of H^+ as well.

We solve for $[H^+]_1$ as a function of the dilute phase concentration $[P^+]_2$. The solution is

$$[H^+]_1 = \frac{2 \times 10^{-4} \times [P^+]_2 + 4 \times 10^{-8}}{4 \times 10^{-4} + [P^+]_1 + [P^+]_2}\, mM \tag{11}$$

and the other quantities follow accordingly.

The solution in the limit of very high partition coefficient ($[P^+]_2 = 0$) is provided below in Supplementary Table 1 assuming room temperature (298 K). The values of the electric potentials as a function of effective partition coefficient, $K = \frac{[P^+]_1}{[P^+]_2}$, are provided in Figure S1.

Supplementary Table S1: Solution to Donnan Equilibrium model in the limit of high partition coefficient

V_{12}	**295.8 mV**
V_{13}	**278.0 mV**
V_{23}	**-17.8 mV**
$[H^+]_1$	**1.99996 × 10^{-12} M** (pH 11.7)
$[Cl^-]_1$	**20.000000002 mM**
$[H^+]_2$	**1.99998 × 10^{-7} M** (pH 6.7)
$[Cl^-]_2$	**1.99998 × 10^{-7} M**
$[H^+]_3$	**1 × 10^{-7} M** (pH 7)
$[Cl^-]_3$	**1 × 10^{-7} M**

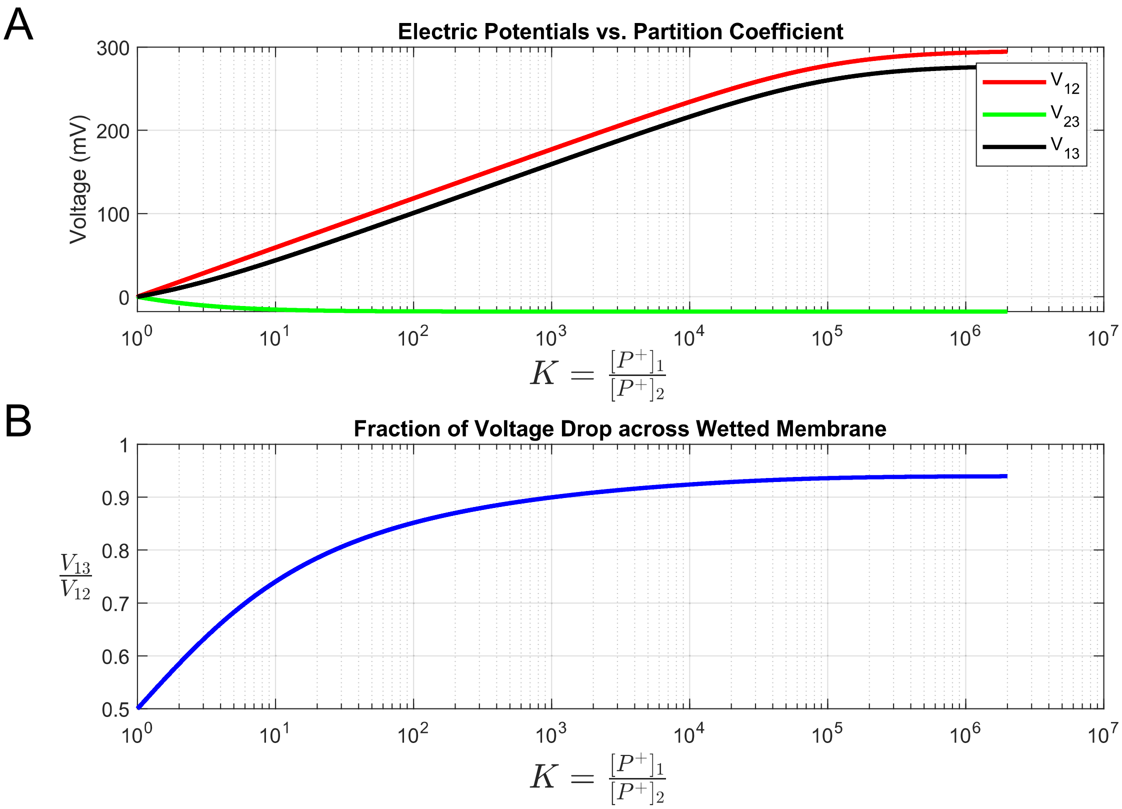

Figure S1: General solution to Donnan Equilibrium model.

Calculation was done holding $[P^+]_1$ = 20 mM fixed, while varying $[P^+]_2$ and solving equation 11. **(A)** Electric Potentials vs. effective partition coefficient, $K = \frac{[P^+]_1}{[P^+]_2}$ **(B)** Fraction of voltage drop which occurs across the wetted membrane region as a function of effective partition coefficient, $K = \frac{[P^+]_1}{[P^+]_2}$.

Supplementary Note S2: Analytical Framework

Consider a 3-component system consisting of a polycation, anion, and water. Briefly, the electrostatic potential is determined by the distribution of charges via the Poisson equation [1]:

$$\nabla^2\psi = -\frac{\rho(\boldsymbol{r})}{\varepsilon} \tag{12}$$

where $\rho(\boldsymbol{r}) = \sum_{i=1}^{3} z_i e n_i(\boldsymbol{r})$ is the charge density, and z_i is the valence and e is the elementary charge. The free-energy functional is given by:

$$F[n_i,\psi] = \int f_{FH}(n_i) + \frac{\kappa_i}{2}\left(\nabla n_i(\boldsymbol{r})\right)^2 + \rho(\boldsymbol{r})\psi(\boldsymbol{r}) \tag{13}$$

where n_i is the local concentration of component i, ψ is the electric potential, κ_i is the gradient cost for component i, and $f_{FH}(n_i)$ is the Flory-Huggins free energy given by:

$$\frac{f_{FH}(n_i)}{k_B T} = \sum_{i=1}^{3} n_i \, ln \, v_i n_i(\boldsymbol{r}) + \sum_{i<j} \chi_{ij} n_i(\boldsymbol{r}) n_j(\boldsymbol{r}) \tag{14}$$

where v_i is the molecular volume of component i, and χ_{ij} is the Flory-Huggins interaction parameter between components i and j. The electrochemical potential is then given by:

$$\mu_i(\boldsymbol{r}) = \frac{\delta F}{\delta n_i} = \frac{\partial F}{\partial n_i} - \nabla \cdot \frac{\partial F}{\partial \nabla n_i} = \frac{\partial f_{FH}}{\partial n_i} - \kappa \nabla^2 n_i(\boldsymbol{r}) + z_i e \psi(\boldsymbol{r}) \tag{15}$$

where

$$\frac{\partial f_{FH}}{\partial n_1} = k_B T \Big[ln\big(v_1 n_1(\boldsymbol{r})\big) + 1 + \frac{v_1}{v_3}\big(1 - ln\big(v_1 n_1(\boldsymbol{r})\big)\big) + \chi_{12} n_2(\boldsymbol{r}) + \chi_{13} n_3(\boldsymbol{r}) - \frac{v_1}{v_3}\big(\chi_{13} n_1(\boldsymbol{r}) + \chi_{23} n_2(\boldsymbol{r})\big)\Big] \tag{16}$$

$$\frac{\partial f_{FH}}{\partial n_2} = k_B T \Big[ln\big(v_2 n_2(\boldsymbol{r})\big) + 1 + \frac{v_2}{v_3}\big(1 - ln\big(v_2 n_2(\boldsymbol{r})\big)\big) + \chi_{12} n_1(\boldsymbol{r}) + \chi_{23} n_3(\boldsymbol{r}) - \frac{v_2}{v_3}\big(\chi_{13} n_1(\boldsymbol{r}) + \chi_{23} n_2(\boldsymbol{r})\big)\Big] \tag{17}$$

Note that the concentration of component 3 is determined by conservation of volume fraction:

$$v_3 n_3(\boldsymbol{r}) = 1 - v_1 n_1(\boldsymbol{r}) - v_2 n_2(\boldsymbol{r}) \tag{18}$$

To determine the equilibrium concentration profiles, and equilibrium electric potential profile we solve the following equation [2]:

$$\frac{dn_i(\boldsymbol{r})}{dt} = -\mu_i(\boldsymbol{r}) + \langle \mu_i(\boldsymbol{r}) \rangle \tag{19}$$

with the boundary conditions:

$$\left.\frac{dn_i}{dx}\right|_{x=0,N} = 0; \left.\frac{d\psi}{dx}\right|_{x=0} = -\frac{\sigma}{\varepsilon}; \left.\frac{d\psi}{dx}\right|_{x=N} = \frac{\sigma}{\varepsilon} \tag{20}$$

which corresponds to a charged boundary with surface charge density σ at $x = 0$, N where $\varepsilon = \varepsilon_0\varepsilon_w$. Note, in general $\varepsilon_0\varepsilon_m \left.\frac{d\psi}{dx}\right|_{x=0} - \varepsilon_0\varepsilon_w \left.\frac{d\psi}{dx}\right|_{x=0} = \sigma$, but we neglected the term proportional to ε_m by assuming $\varepsilon_m \ll \varepsilon_w$. The surface charge density is determined by

$$\sigma = \frac{e}{A_L} f[\theta(z-1) - (1-\theta)] \tag{21}$$

where e is the elementary charge, A_L is the area per lipid, f is the fraction of charged lipids, and θ is the fractional occupancy of binding sites which is determined by the Langmuir Isotherm

$$\theta = \frac{K_{p+} n_{p+}\big|_{x=0,N}}{1 + K_{p+} n_{p+}\big|_{x=0,N}} \tag{22}$$

where K_{p+} is the polycation-membrane binding constant. To account for the change in electrochemical potential at the membrane due to binding, we introduce a binding energy which is related to the binding constant by

$$\omega = -k_B T \ln(K_{p+}) \tag{23}$$

so that the binding free energy density becomes

$$F_{binding} = \omega n_{b,v} = \frac{\omega n_{b,s}}{dx} \tag{24}$$

where $n_{b,v}$ is the concentration of bound polycations per unit volume, and $n_{b,s}$ is the concentration of bound polycations per unit area.

The fraction of bound sites can be written as

$$\theta = \frac{n_{b,s}}{f/A_L} \rightarrow n_{b,s} = \frac{\theta f}{A_L} \tag{25}$$

so that

$$F_{binding} = \frac{-k_B T \ln(K_{p+})\, n_{b,s}}{dx} = \frac{-k_B T \ln(K_{p+})\, f}{dx A_L} \frac{K_{p+} n_{p+}\big|_{x=0}}{1 + K_{p+} n_{p+}\big|_{x=0}} \tag{26}$$

and finally,

$$\mu_1(0) = \cdots + \frac{\partial F_{binding}}{\partial n} = \cdots - \frac{f K_{p+} k_B T \ln(K_{p+})}{A_L dx (K_{p+} n_{p+}\big|_{x=0} + 1)^2} \tag{27}$$

which we incorporate into the model.

We solve equation 19 with the appropriate boundary conditions (eq. 20) by discretizing the spatial derivatives using central finite differences, and integrate the time derivatives using MATLAB's ode15s() function. All code used in this paper will be made available at (https://github.com/Deniz-Lab/Biomolecular-Condensates-Local-Membrane-Potentials)

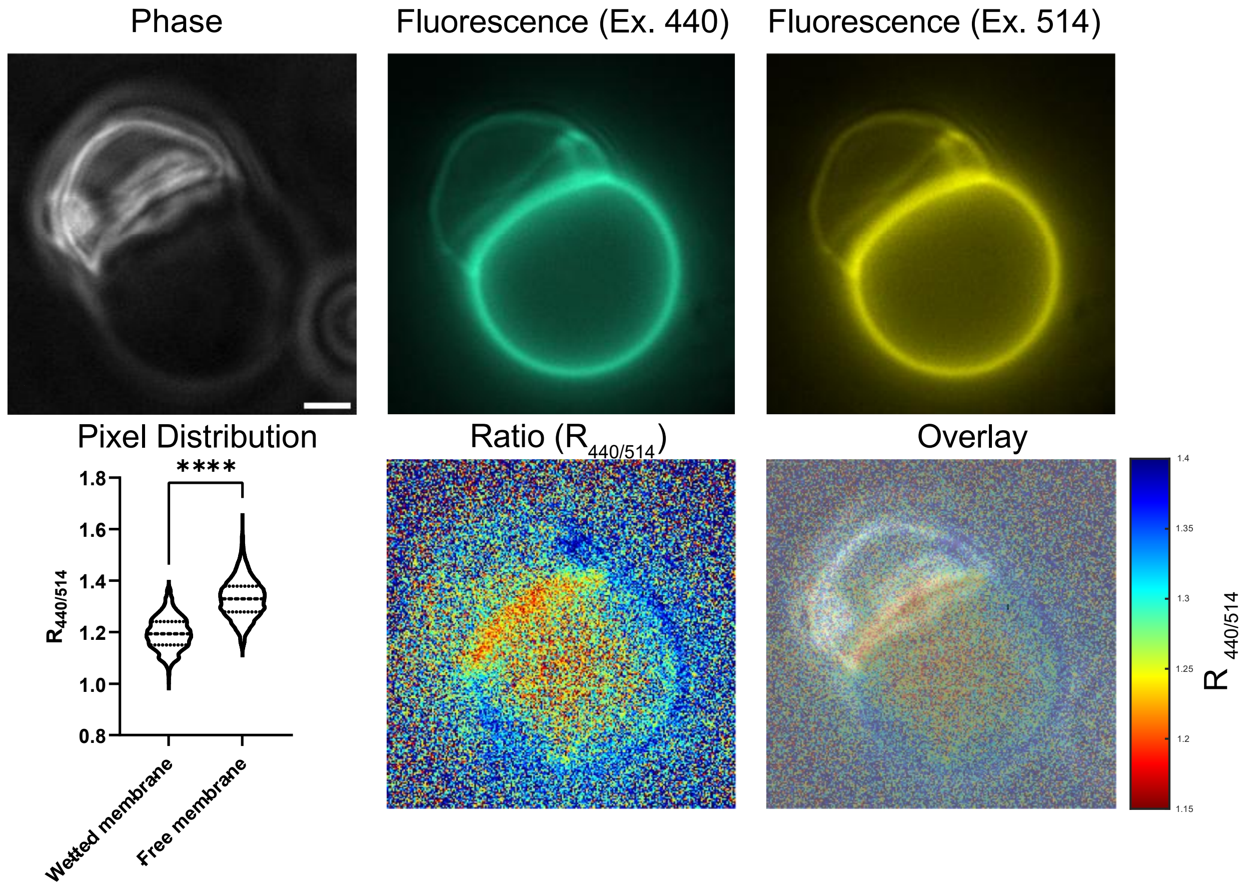

Figure S2: Main-text Figure 2 without thresholding. Same as main-text Figure 2, but raw ratiometric images are not thresholded. See main-text Figure 2 for details.

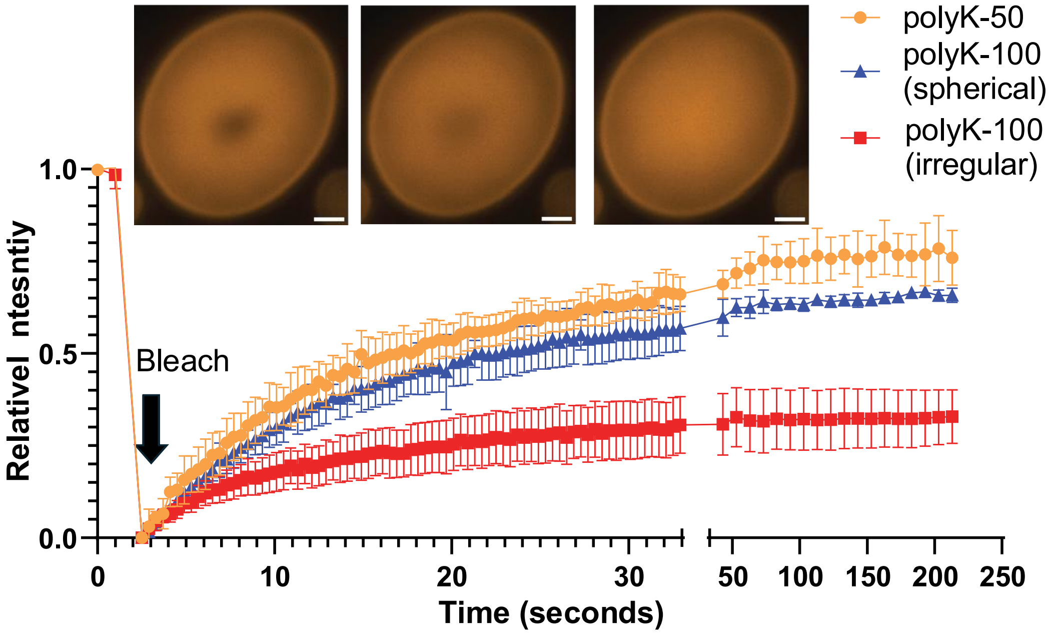

Figure S3: FRAP (Fluorescence Recovery After Photobleaching) measurements. FRAP curves of condensates corresponding to 30 µM polyK-100 1.275 mM ATP, 2.5 mM $MgCl_2$, 50 mM Tris-HCl pH 7.5 or 60 µM polyK-50 1.275 mM ATP, 2.5 mM $MgCl_2$, 50 mM Tris-HCl pH 7.5 (polyK-50, n = 5; polyK-100 irregular, n = 5; polyK-100 spherical, n = 2). Inset images correspond to polyK-50 condition.

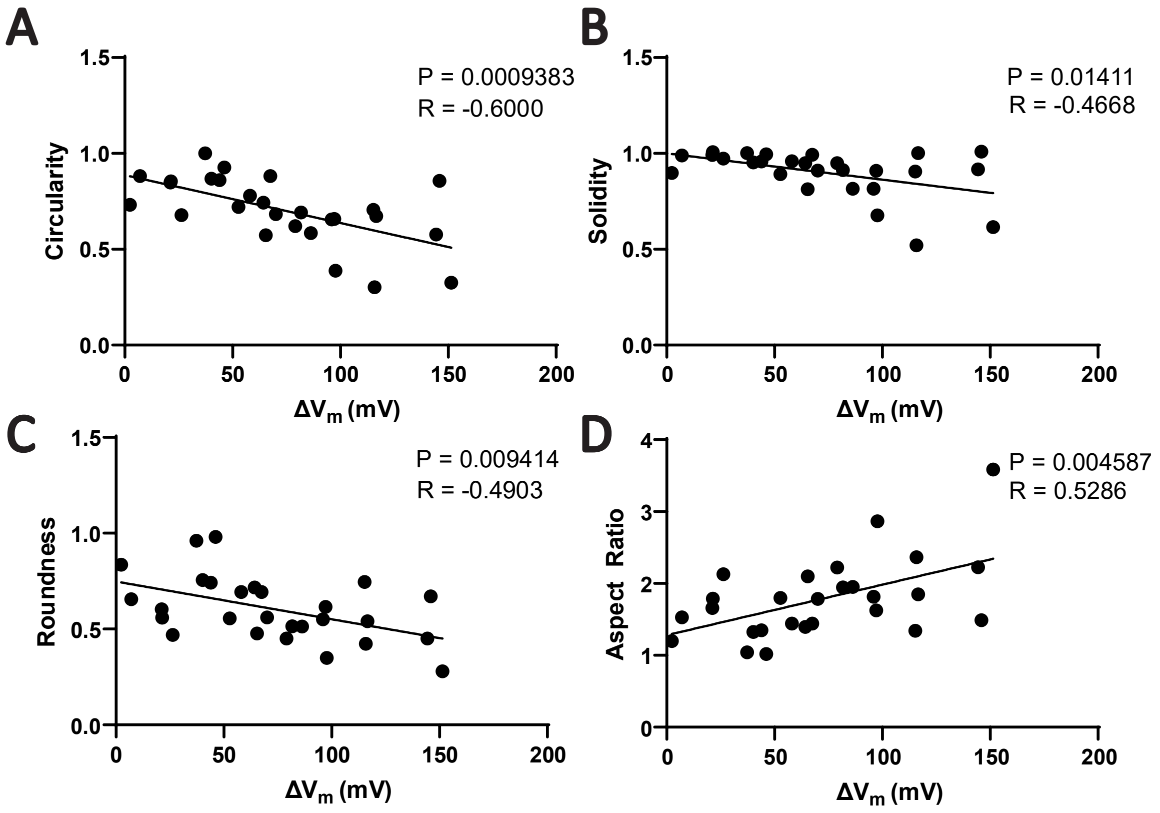

Figure S4: Correlation of condensate shape parameters with ΔV_m. Condensates (20 µM polyK-50, 20 µM polyK-100, 1 mM ATP, 2.5 mM $MgCl_2$, 500 mM glucose, 50 mM Tris-HCl pH 7.5). R value is Pearson's correlation coefficient (n = 27). **(A)** Circularity vs. ΔV_m **(B)** Solidity vs. ΔV_m **(C)** Roundness vs. ΔV_m **(D)** Aspect Ratio vs. ΔV_m Definitions: Circularity = $\frac{4\pi \times \text{Area}}{perimeter^2}$; Solidity = $\frac{Area}{Convex\ Area}$; Roundness = $\frac{4 \times \text{Area}}{\pi \times Major\ Axis^2}$; Aspect Ratio = $\frac{Major\ Axis}{Minor\ Axis}$

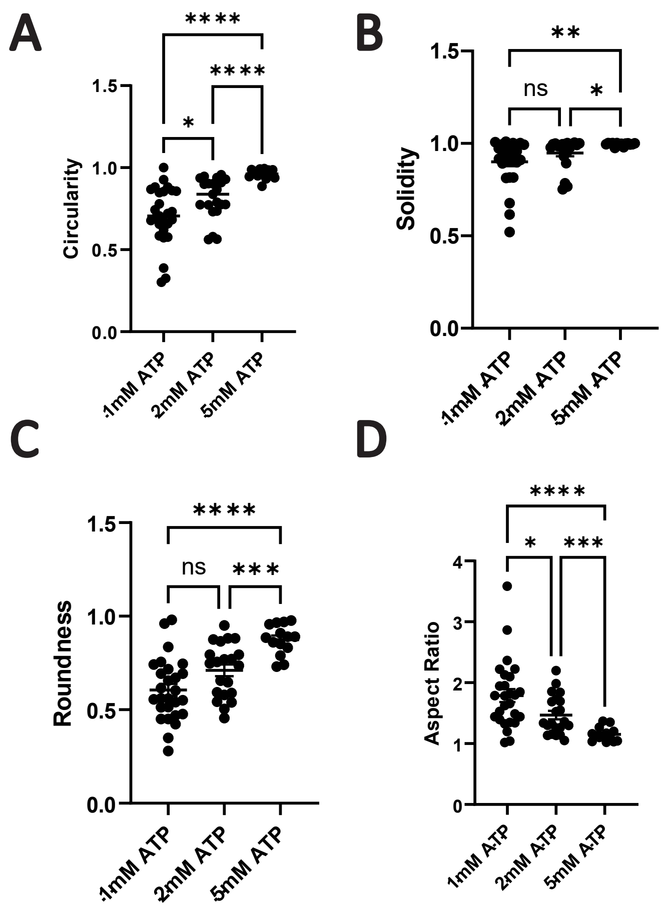

Figure S5: Condensate shape parameters across different ATP concentrations. Condensates (20 µM polyK-50, 20 µM polyK-100, x mM ATP, 2.5 mM $MgCl_2$, 500 mM glucose, 50 mM Tris-HCl pH 7.5). One-way ANOVA and Dunnett's T3 multiple comparisons test ($p<0.0001$,**** | $p<0.001$,*** | $p<0.01$,** | $p<0.05$,*). ($n = 27, 21, 14$). **(A)** Circularity vs. ΔV_m **(B)** Solidity vs. ΔV_m **(C)** Roundness vs. ΔV_m **(D)** Aspect Ratio vs. ΔV_m Definitions: Circularity $= \frac{4\pi \times Area}{perimeter^2}$; Solidity $= \frac{Area}{Convex\ Area}$; Roundness $= \frac{4 \times Area}{\pi \times Major\ Axis^2}$; Aspect Ratio $= \frac{Major\ Axis}{Minor\ Axis}$

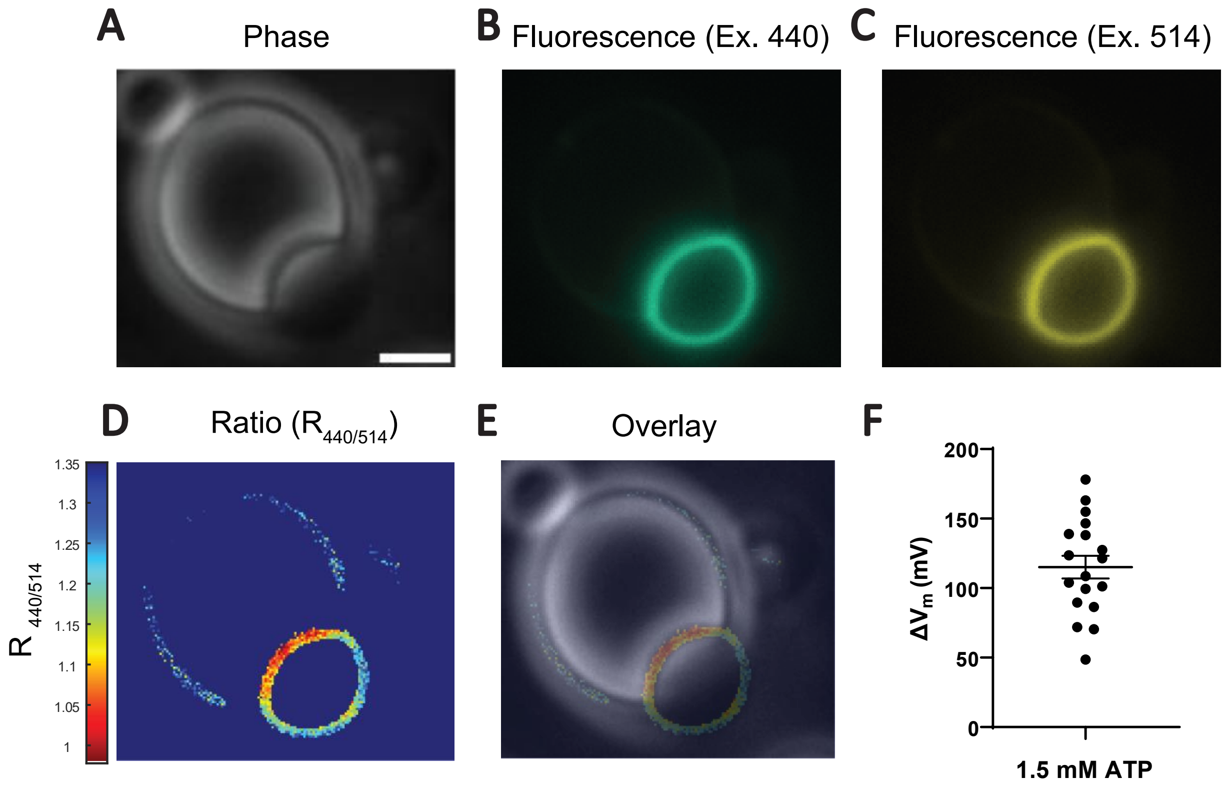

Figure S6: poly(RG)/ATP condensate – GUV measurements. 40% PA, 60% POPC GUVs (0.4 wt% poloxamer188 included in inner solution) with poly(RG)/ATP condensates (2 mg/mL (RG)-15, 1.5 mM ATP, 500 mM glucose, 50 mM Tris-HCl pH 7.5). **(A)** Phase contrast image of condensate wetting GUV. **(B, C)** Fluorescence images of GUV membrane labeled by di-8-anepps, with 440 nm and 514 nm excitation respectively. **(D)** Thresholded raw ratiometric image, **(E)** Overlay of phase contrast image and ratiometric image. **(F)** Summary figure (n = 18). Scale bar is 2 µm.

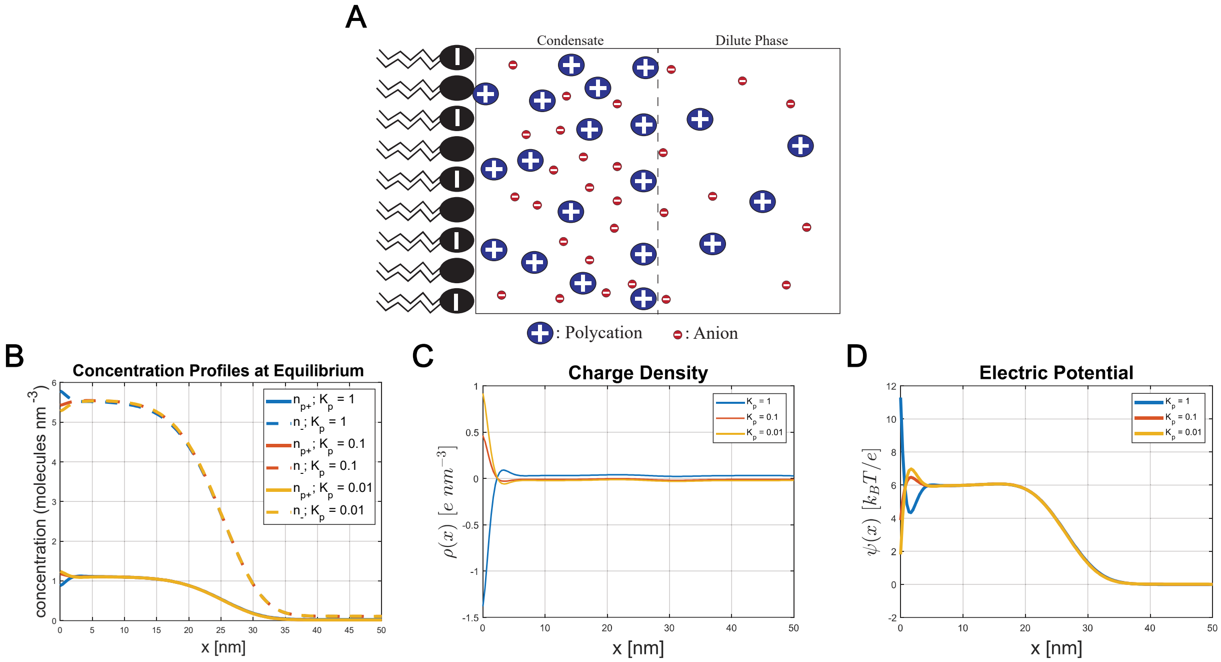

Figure S7: Effect of varying binding constant K_{p+} in numerical simulation of a condensate in the presence of a charged membrane. (p+) indicates the polycation while (-) indicates the anion. Parameters used for calculation: $\kappa_{p+} = 100k_BT$; $\kappa_- = 20k_BT$; $\chi_{12} = -1.0$; $\chi_{13} = 1.5$; $\chi_{23} = -0.09$; $N1 = 20$; $N2 = 1$; $N3 = 1$; $v = 0.03\ nm^3$; $z1 = 5$; $z2 = -1$; $z3 = 0$; $A_L = 0.5\ nm^2$; $f = 0.5$. See Supplementary Note 2 for details. **(A)** Schematic illustration of model geometry **(B)** Equilibrium concentration profiles. Region with high concentrations corresponds to the dense phase (condensate), region with low concentrations corresponds to the dilute phase. **(C)** Equilibrium charge density. **(D)** Equilibrium Electric Potential.

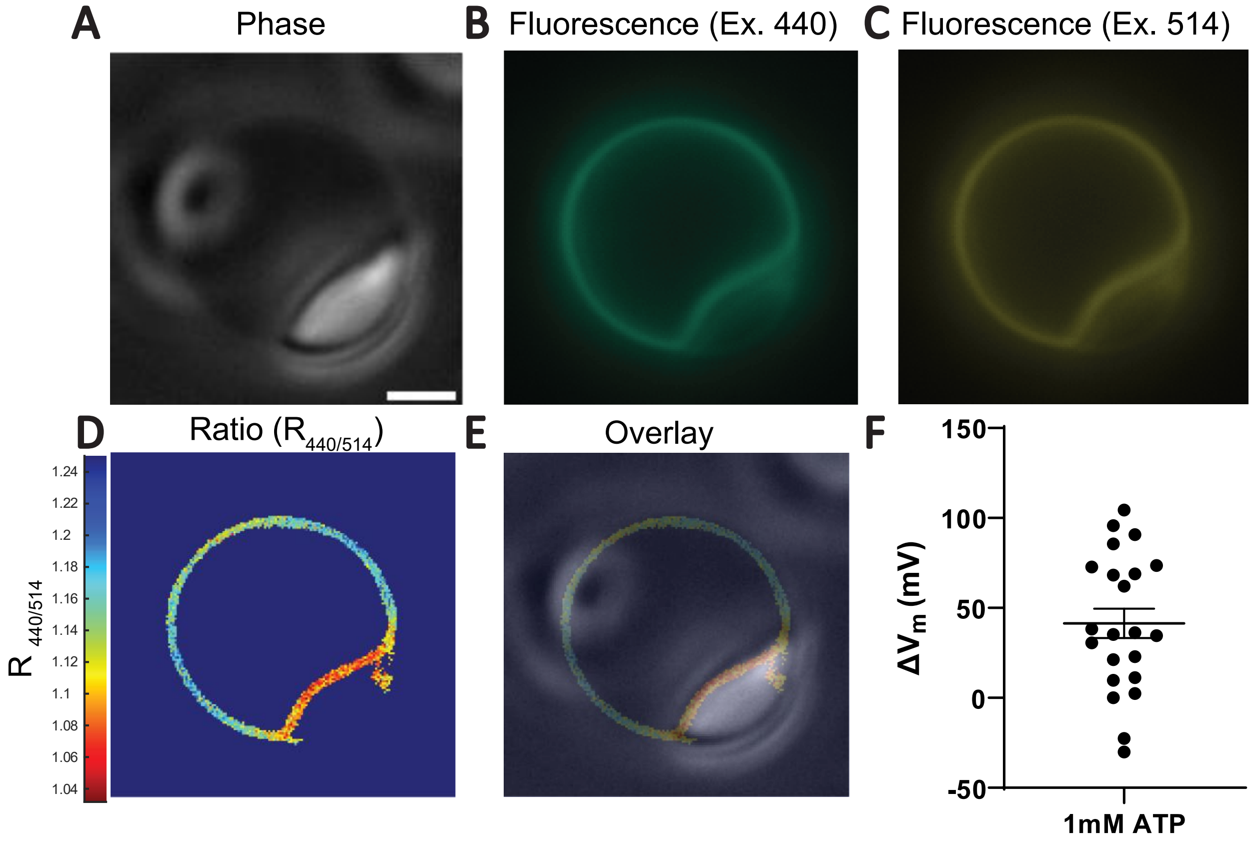

Figure S8: DOPC/PA GUV control. 10% PA, 90% DOPC GUVs (0.4 wt% poloxamer188 included in inner solution) with polyK/ATP condensates (20 µM polyK-50, 20 µM polyK-100, 1 mM ATP, 2.5 mM $MgCl_2$, 500 mM glucose, 50 mM Tris-HCl pH 7.5). **(A)** Phase contrast image of condensate wetting GUV. **(B, C)** Fluorescence images of GUV membrane labeled by di-8-anepps, with 440 nm and 514 nm excitation respectively. **(D)** Thresholded raw ratiometric image, **(E)** Overlay of phase contrast image and ratiometric image. **(F)** Summary figure (n = 22). Scale bar is 2 µm.

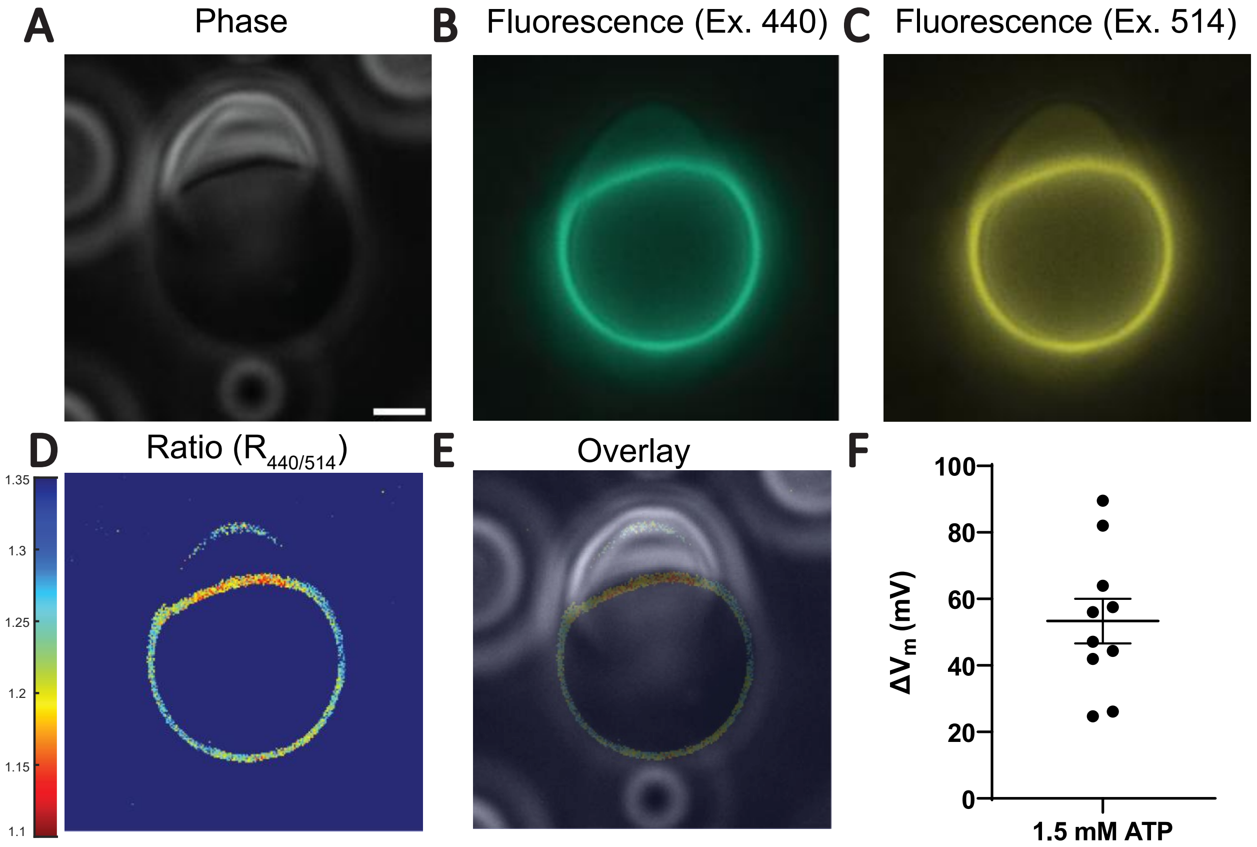

Figure S9: POPC/POPS GUV control. 50% POPS, 50% POPC GUVs (0.4 wt% poloxamer188 included in inner solution) with polyK/ATP condensates (20 µM polyK-50, 20 µM polyK-100, 1.5 mM ATP, 2.5 mM $MgCl_2$, 500 mM glucose, 50 mM Tris-HCl pH 7.5). **(A)** Phase contrast image of condensate wetting GUV. **(B, C)** Fluorescence images of GUV membrane labeled by di-8-anepps, with 440 nm and 514 nm excitation respectively. **(D)** Thresholded raw ratiometric image, **(E)** Overlay of phase contrast image and ratiometric image. **(F)** Summary figure (n = 10). Scale bar is 2 µm.

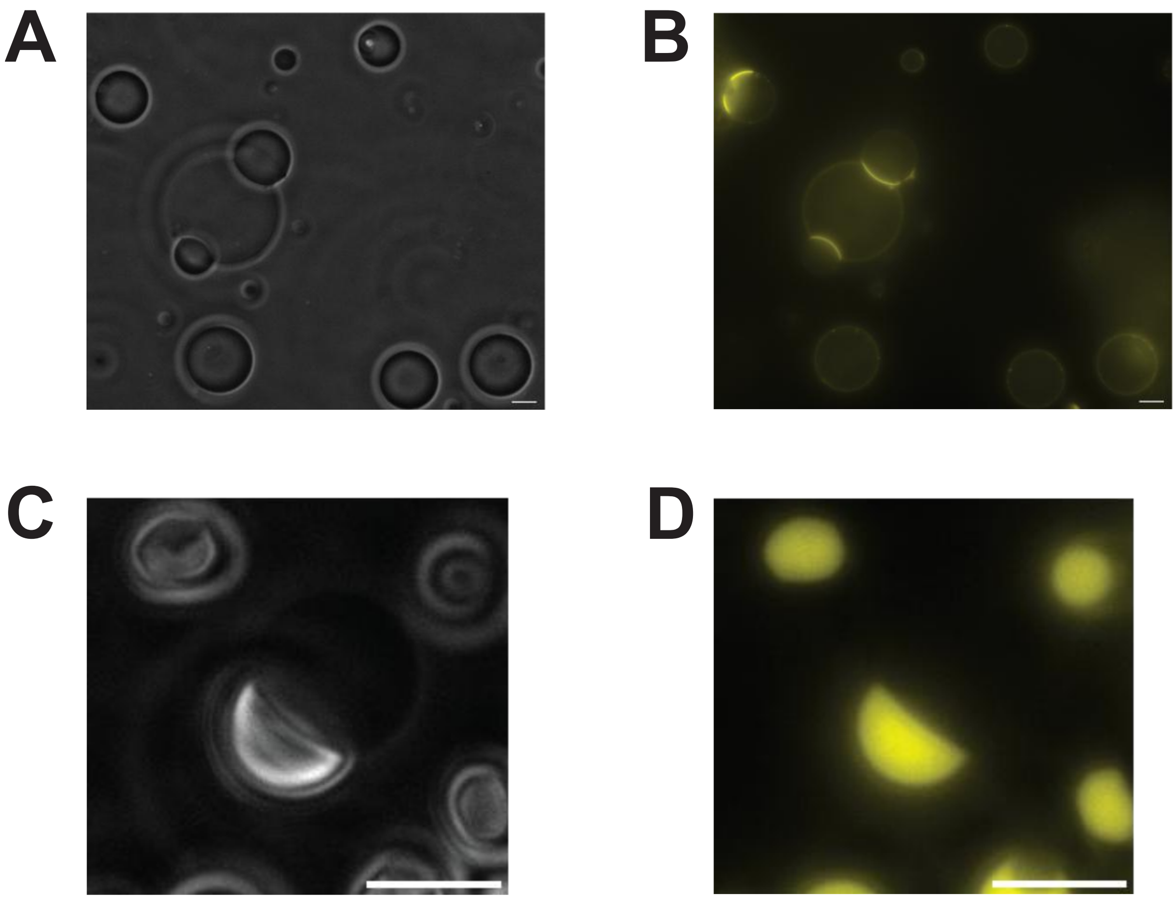

Figure S10: Localization of labeled polyK-Atto565. Top Row: 20% PA, 80% POPC GUVs with 150 nM polyK-Atto565 only. **(A)** Phase contrast image **(B)** Epifluorescence image. Bottom row: condensates are formed with 20 µM polyK-50, 20 µM polyK-100, 1 mM ATP, 2.5 mM $MgCl_2$, 500 mM glucose, 50 mM Tris-HCl pH 7.5, and 150 nM polyK-Atto565. **(C)** Phase contrast image **(D)** Epifluorescence image. Scale bar is 5 µm.

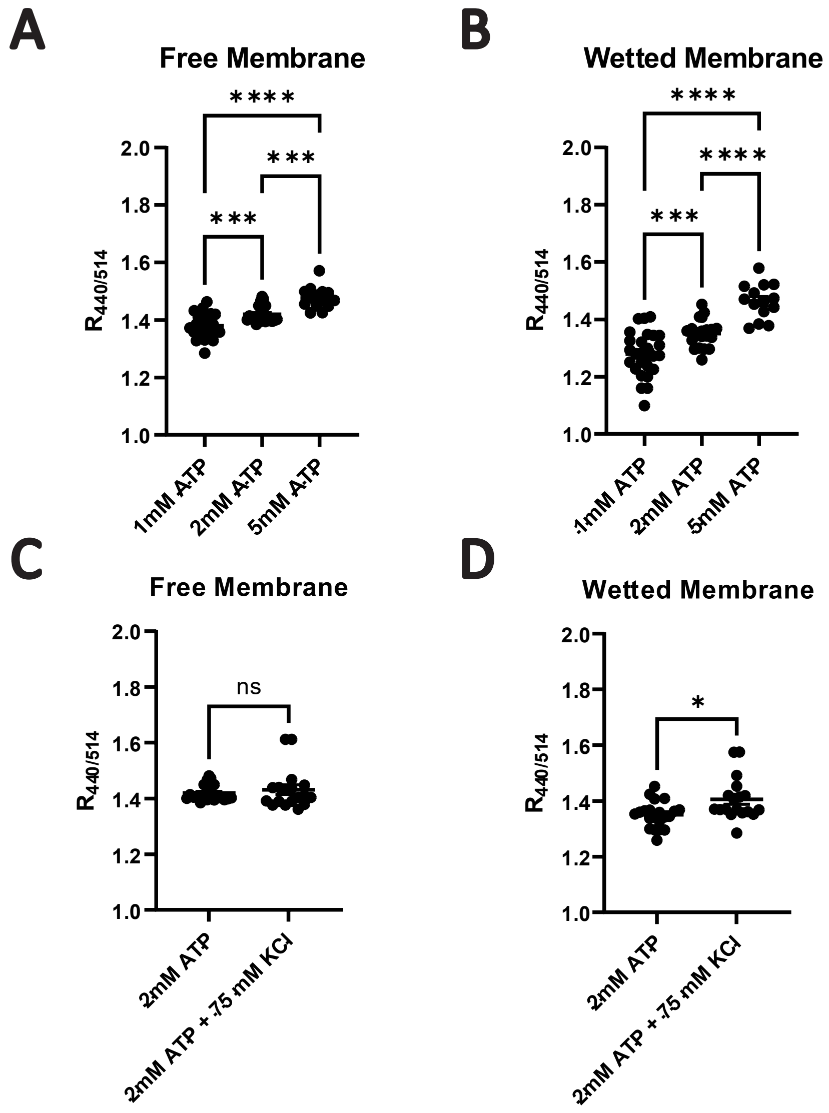

Figure S11: $R_{440/514}$ values for membrane regions used to calculate ΔV_m in Figure 3.

(A) Free Membrane Region $R_{440/514}$ with varying ATP concentration (n = 27, 21, 14) **(B)** Wetted Membrane Region $R_{440/514}$ with varying ATP concentration (n = 27, 21, 14) **(C)** Free Membrane Region $R_{440/514}$ with varying salt concentration (n = 21, 18) **(D)** Wetted Membrane Region $R_{440/514}$ with varying salt concentration (n = 21, 18). Statistical analysis is One-way ANOVA and Dunnett's T3 multiple comparisons test ($p<0.0001$,**** | $p<0.001$,*** | $p<0.05$,*)

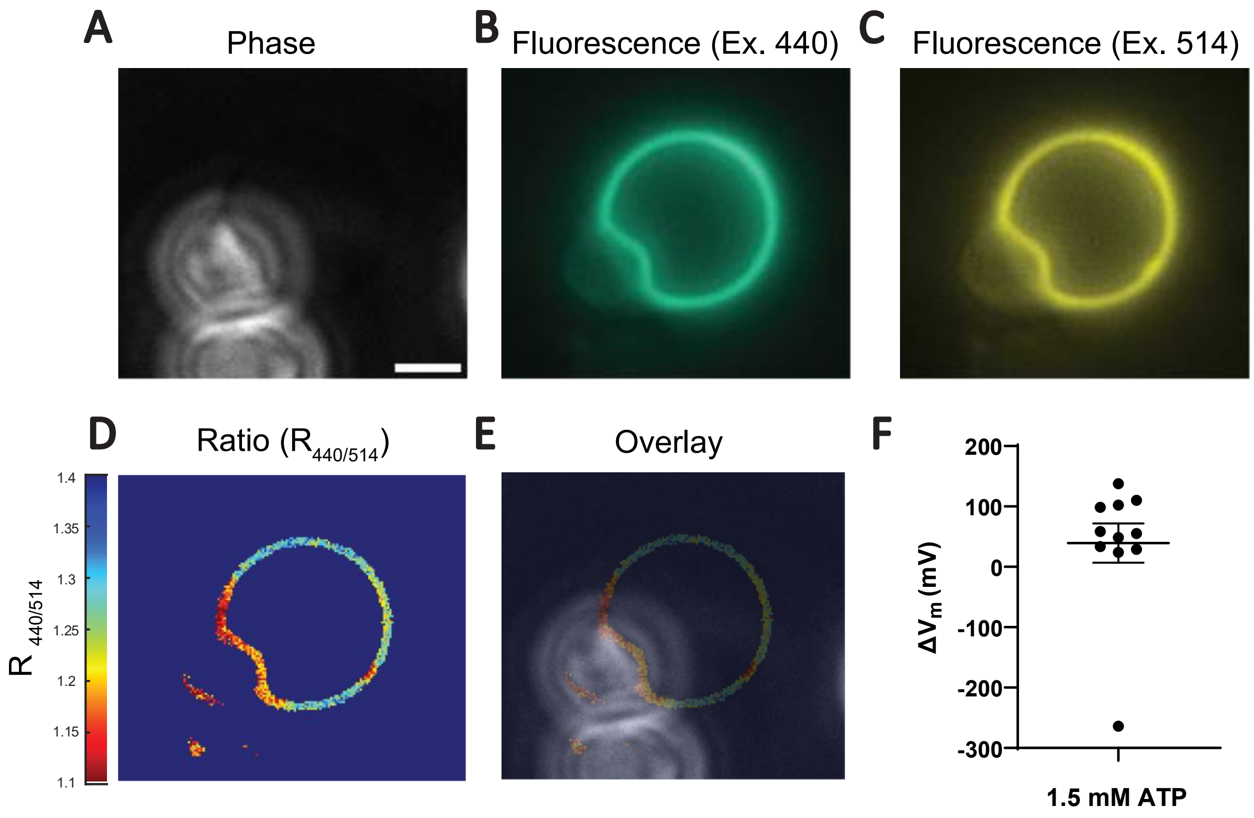

Figure S12: Electroformation control. 20% PA, 80% POPC GUVs with polyK/ATP condensates (20 µM polyK-50, 20 µM polyK-100, 1.5 mM ATP, 2.5 mM $MgCl_2$, 110 mM glucose, 50 mM Tris-HCl pH 7.5). **(A)** Phase contrast image of condensate wetting GUV. **(B, C)** Fluorescence images of GUV membrane labeled by di-8-anepps, with 440 nm and 514 nm excitation respectively. **(D)** Thresholded raw ratiometric image, **(E)** Overlay of phase contrast image and ratiometric image. **(F)** Summary figure (n = 11). Scale bar is 2 µm.

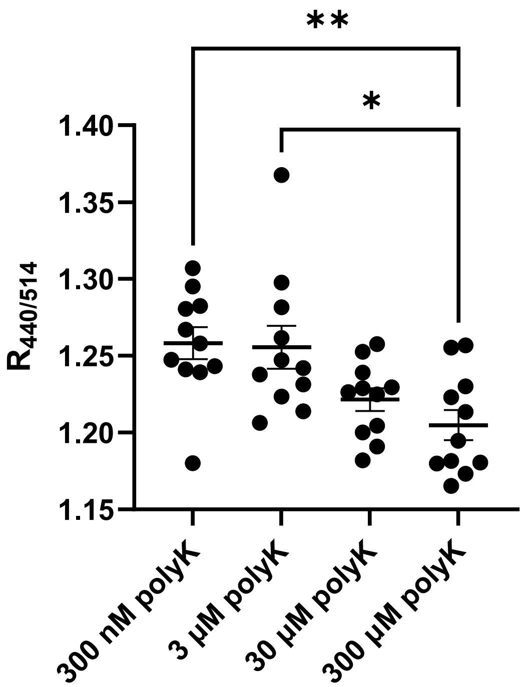

Figure S13: polyK titration. 20% PA, 80% POPC GUVs (500 mM glucose, 50 mM Tris-HCl), with addition of polyK-100 at various concentrations. Each point represents $R_{440/514}$ calculated by segmenting all GUVs in a field of view and taking the ratio of the average intensity in the 440 nm and 514 nm channel (n = 11). Statistical analysis is One-way ANOVA and Dunnett's T3 multiple comparisons test (p<0.01,** | p<0.05,*).

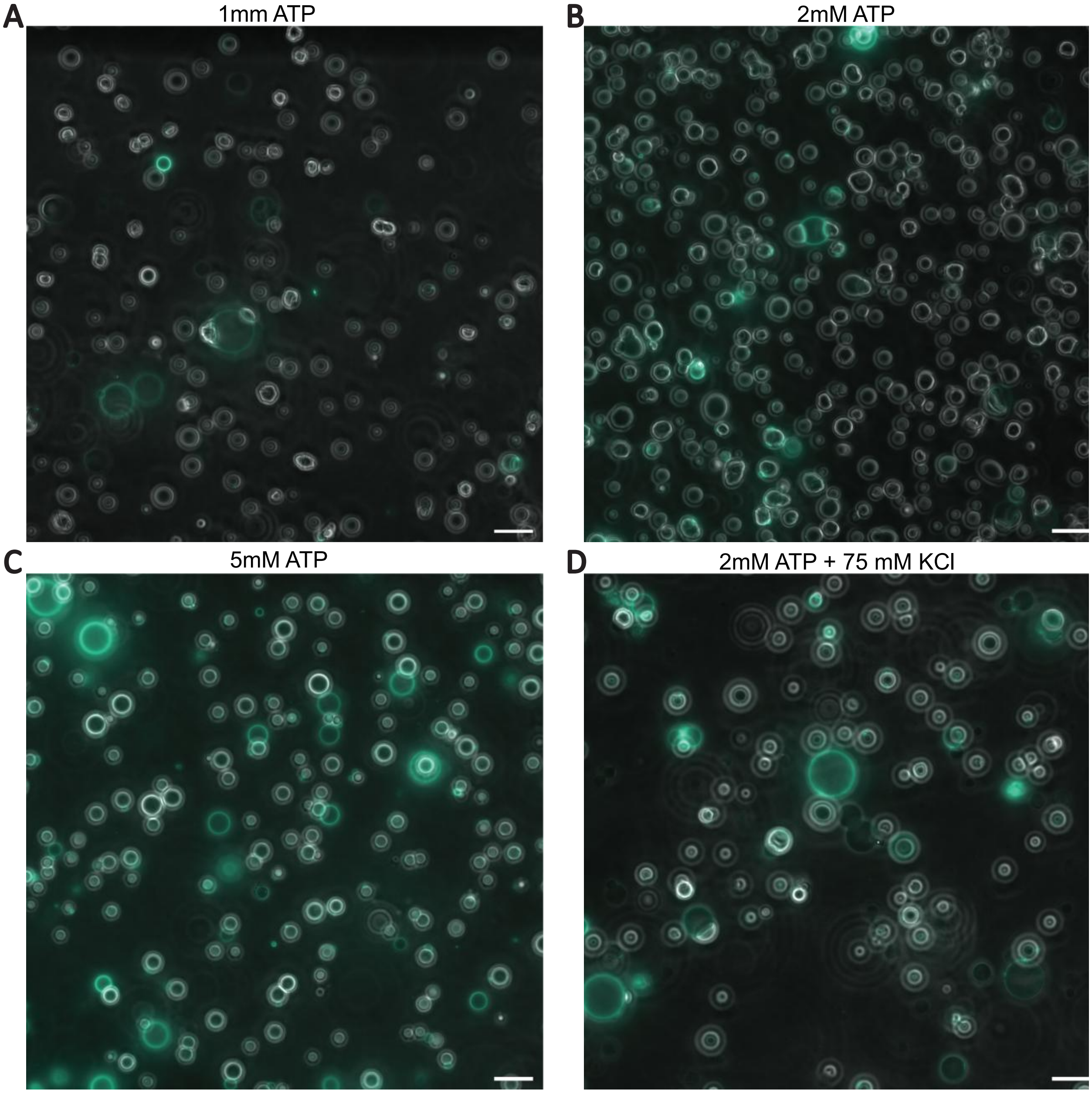

Figure S14: Full fields of view. Full fields of view corresponding to panels from main-text Figure 3. Scale Bar = 10 μm.

Supplementary Materials and Methods

1-palmitoyl-2-oleoyl-sn-glycero-3-phosphocholine (POPC), 1-palmitoyl-2-oleoyl-sn-glycero-3-phospho-L-serine (POPS), 1,2-dioleoyl-sn-glycero-3-phosphocholine (DOPC), and 1,2-dioleoyl-sn-glycero-3-phosphate (PA) were purchased from Avanti Polar Lipids (Alabaster, AL). 4-[2-[6-(dioctylamino)-2-naphthalenyl]ethenyl]-1-(3-sulfopropyl)-pyridinium (di-8-anepps) was purchased from Cayman Chemical (Ann Arbor, Michigan). Mineral Oil (USP grade) was purchased from Target (Up & Up). Poly-L-lysine hydrochloride (polyK) was purchased from Alamanda Polymers (Huntsville, AL). ATP disodium salt hydrate (587.17 Da) was purchased from MedChemExpress (Monmouth Junction, NJ). RG-15 (arginine-glycine)$_{15}$ was ordered and custom synthesized from GenScript (Piscataway, NJ). ATTO 565 NHS-ester was purchased from ATTO-TEC (Siegen, Germany). Poloxamer 188 was purchased from Oakwood Products (West Columbia, South Carolina).

<u>*Sample Preparation*</u>

The Inverted emulsion method was used to generate Giant Unilamellar Vesicles (GUVs) [3]. Briefly, lipids from stock solutions in chloroform (10 mg/mL) were dissolved in mineral oil for a final concentration of 0.6 mg/mL and heated at 80°C in a fume hood to evaporate the chloroform.

First, 200 µL outer aqueous solution (50 mM Tris-HCl (pH 7.5), 500 mM Glucose) was added to a 1.5 mL microcentrifuge tube. In a separate 2 mL microcentrifuge tube, 50 µL inner aqueous solution (50 mM Tris-HCl (pH 7.5), 500 mM Sucrose) was combined with 400 µL of lipids in oil (0.15 mg/mL lipid) and vortexed on high for 40-60 seconds to create an emulsion. Note that for Figures S6, S8, and S9 0.4 wt% poloxamer 188 was added to the inner solution to improve vesicle yield and reduce aggregation. The emulsion was then gently pipetted on top of the bottom aqueous solution and incubated at room temperature for 20 minutes to equilibrate the oil-water interface. The tube was then centrifuged at 16000 g for 15 minutes, producing a pellet of vesicles. The oil layer and part of the aqueous layer was pipetted off, and the pellet were resuspended in 100 µL of bottom aqueous solution. The 100 µL vesicle suspension was then combined with 400 µL of bottom aqueous solution in a new 1.5 mL microcentrifuge tube and centrifuged at 5000 g for 5 minutes. Part of the bottom aqueous solution was removed, and the pellet was resuspended in a final volume of 100-200 µL. For imaging, 4 µL of the GUV suspension was added to a coverslip followed by 1 µL of dye solution (10 µM di-8-anepps, 50 mM Tris-HCl (pH 7.5), 500 mM Glucose) and 5 µL of preformed condensates (in 500 mM glucose, 50 mM Tris-HCl (pH 7.5). The condensate buffer also included 2.5 mM $MgCl_2$ to approximate the physiological state of ATP in the cell (Mg-ATP), and pH 7.5 was also chosen to approximate physiological conditions. The preformed condensates were made at 2X concentration and incubated in 0.65 mL tubes for 30 min prior to imaging. For Figures S6, S8 S11, and S13, the GUVs were pre-incubated with 1 µM di-8-anepps for 1 hour and then washed prior to imaging. Coverslips were passivated with 10-20 mg/mL BSA for at least 24 hours and rinsed with DI water prior to imaging.

We noticed that the ATP concentrations of liquid stocks made by weighing out ATP were consistently 15% lower than the ATP concentrations determined by measuring the absorbance at

260 nm on a NanoDrop2000c, using an extinction coefficient of 15,400 cm^{-1} M^{-1}. We corrected our ATP concentrations to match the concentration determined using the absorbance.

Imaging and Analysis

Epifluorescence microscopy was performed on a Nikon Ti2-E automated inverted microscope with a 100x oil immersion objective (Plan Apo λ 100x Oil Ph3 DM) and a Kinetix sCMOS camera (Teledyne Photometrics). Images were acquired with the NIS Elements software version 5.42.03. To generate ratiometric pixel distributions, ROIs were drawn in MATLAB, and the list of individual pixel intensity ratios for each ROI was exported to GraphPad Prism for graphing and statistics. For ratiometric images, the dynamic range of the ratios was reduced for visualization purposes by setting cutoff maximum and minimum ratios and assigning values greater or less than those cutoffs to the maximum or minimum ratio respectively. For the ratiometric image in Figure 2, the noisy background was set to a constant (background) value by using local intensity thresholds determined by adaptive window sizes based on the local intensity gradient. All code used in this paper will be made available at (https://github.com/Deniz-Lab/Biomolecular-Condensates-Local-Membrane-Potentials). Phase contrast images were taken with 100 ms exposure and a Nikon DAPI 96360 filter cube. Epifluorescence images were taken using 440 nm and 514 nm excitation with 20-30 ms exposure and a Nikon Spectra-Triple filter cube (Emission 464 nm - 486 nm, 532 nm - 554 nm, 603 nm – 800 nm). Note, di-8-anepps emission spectrum spans from ~ 500 nm – 850 nm. Image resolution was 15.14 pixels per micron.

To generate summary results/figures, the following disqualifying criteria were used to curate data prior to processing: 1. No clear membrane segment between condensate and membrane; 2. Signal to background ratio less than 3 in the 440 nm channel; 3. Condensate moved between frames; 4. GUV or condensate out of focus; 5. Background artifacts near vesicle; 6. GUV diameter < 2µm; 7. Pixel Saturation; 8. Complete wetting of GUV (no free membrane segment) 9. GUV cut off at edge of field of view. 10. Other uninterpretable features. When measuring ROIs we also followed two additional rules. 1. If more than one condensate or GUV is contacting the GUV of interest, measure the union of the free membrane segments as the "free membrane". 2. If another vesicle is adsorbed to the GUV of interest, do not measure the membrane segment at the interface of the two vesicles.

In ImageJ, polygonal ROIs were drawn corresponding to the membrane segment wetted by the condensate, the free membrane segment, the background within the condensate, and the background adjacent to the free membrane (outside the GUV). The mean intensity for all ROIs were exported to Microsoft Excel, and the final background subtracted intensities were calculated as ($I_{wetted\ membrane}$ - $I_{condensate\ background}$) and ($I_{free\ membrane}$ –$I_{free\ background}$). Using the background subtracted intensities, we calculated the excitation ratios $R_{440/514}$ for the "free membrane" and "wetted membrane" segments. We then calculated the percent change in $R_{440/514}$ using the equation, $\%\ (\Delta R/R) = 100 \times \frac{R_{free} - R_{wetted}}{R_{free}}$, and this quantity was subsequently converted to change in voltage using the equation $\Delta V_m = \frac{100\ mV}{10\ \%} \times \%\ (\Delta R/R)$. The conversion factor 10%/100 mV has been commonly used in the literature [4, 5].

All statistical analysis was done in GraphPad Prism.

FRAP

For FRAP (Fluorescence recovery after photobleaching) experiments, 300 nM polyK100-Atto565 was doped into the polyK/ATP coacervates. The bleach radius was set to 0.2 µm in the center of the droplet, The stimulation dwell time was set to 100 µs, and stimulation was done with 405 nm (20% intensity), and 561 nm (40% intensity) lasers. Acquisition was at 561 nm with 50 ms exposure. Bleached droplets were around 4-8 microns in diameter. FRAP curves were calculated using the equation $I_N(t) = \frac{I(t)-I_0}{R(t)-I_0}$ where I_N is the normalized intensity, $I(t)$ is the raw intensity of the bleached ROI at time t, I_0 is the raw intensity immediately after bleaching, and $R(t)$ is the raw intensity of a reference droplet at time t.

Galvani Potential Measurements

Glass capillary electrodes were made by filling Pasteur pipettes with 3M KCl, 1% agarose. Ag/AgCl wires were inserted into the pipettes, and connected to a multimeter (BTMETER, BT-90EPD) with data logging capability. 10 mL of condensate solution was prepared in 15 mL conical tubes and spun down using a swinging-bucket rotor at 4000g to produce a visible white layer at the bottom of the tube. Both electrodes were initially placed in the dilute (upper) phase, then one electrode was inserted into the dense (bottom) phase to measure the Galvani potential and then removed back into the dilute phase. This was repeated three times per sample, but only the first measurement was used for the subsequent analysis.

Electroformation

30 µL of a 1 mg/mL solution of lipids in chloroform was deposited onto an ITO glass slide using a Hamilton syringe and dried in the fume hood for 30 minutes. A silicone isolator with double sided adhesive was sandwiched between the two ITO slides to create the electroformation chamber, and the lipids were rehydrated in a 200 mM sucrose solution. Immediately, the ITO slides were connected to a function generator and electroformation was done at 1.1 V, 10 Hz for 2 hours at room temperature. The vesicles were then collected and washed in a buffer consisting of 110 mM glucose, 50 mM Tris-HCl (pH 7.5).

polyK labeling

We followed a labeling protocol from Thermo Scientific for NHS-Fluorescein (https://assets.thermofisher.com/TFS-Assets/LSG/manuals/MAN0011647_NHSFluorescein_UG.pdf) and adapted it for labeling polyK-100 with ATTO 565 with minor modifications. Briefly, we incubated polyK-100 with ATTO 565 NHS-ester at a 1:1 molar ratio for 1 hour in the dark at room temperature in a 20 mM HEPES (pH 8.2) buffer. We then removed excess dye by passing the sample through a Pierce™ Dye Removal Column (ThermoFisher catalog no. A44296). Next, we filtered the sample with an Amicon® Ultra-0.5 Centrifugal Filter (3000 MWCO) to buffer exchange into Milli-Q water. The final stock concentration of polyK-Atto565 was approximately 30 µM, and was stored in the -20°C freezer for future use.

Data Availability

All data, Analysis, and Figures will be made available at https://doi.org/10.5281/zenodo.14968978

SI References

[1] C. A. W. Arghya Majee, Frank Jülicher, Charge separation at liquid interfaces, *Physical Review Research* **2024**, *6*.

[2] A. Onuki, Ginzburg-Landau theory of solvation in polar fluids: Ion distribution around an interface, *Physical Review E* **2006**, *73*.

[3] Y. Matsushita-Ishiodori, M. M. Hanczyc, A. Wang, J. W. Szostak, T. Yomo, Using Imaging Flow Cytometry to Quantify and Optimize Giant Vesicle Production by Water-in-oil Emulsion Transfer Methods, *Langmuir* **2019**, *35*, 2375-2382.

[4] J. Zhang, R. M. Davidson, M.-d. Wei, L. M. Loew, Membrane Electric Properties by Combined Patch Clamp and Fluorescence Ratio Imaging in Single Neurons, *Biophysical Journal* **1998**, *74*, 48-53.

[5] L. Loew, L. Cohen, J. Dix, E. Fluhler, V. Montana, G. Salama, W. Jian-young, A naphthyl analog of the aminostyryl pyridinium class of potentiometric membrane dyes shows consistent sensitivity in a variety of tissue, cell, and model membrane preparations, *The Journal of Membrane Biology* **1992**, *130*, 1-10.
